# Neurologic music therapy for non-fluent aphasia: a systematic review and meta-analysis of randomized controlled trials

**DOI:** 10.3389/fneur.2024.1395312

**Published:** 2024-05-23

**Authors:** Jiayi Gu, Wei Long, Siqin Zeng, Chengjuan Li, Cuini Fang, Xiaoying Zhang

**Affiliations:** ^1^Department of Rehabilitation, Hunan Provincial People’s Hospital, The First Affiliated Hospital of Hunan Normal University, Changsha, China; ^2^Medicine College, Hunan Normal University, Changsha, China; ^3^Department of Rehabilitation, The First Hospital of Changsha, The Affiliated Changsha Hospital of Xiangya School of Medicine, Central South University, Changsha, China; ^4^School of Rehabilitation Medicine, Capital Medical University, Beijing, China; ^5^Music Therapy Center, China Rehabilitation Research Center, Beijing, China

**Keywords:** neurologic music therapy, aphasia, systematic review, meta-analysis, speech function

## Abstract

**Introduction:**

The efficacy of neurologic music therapy (NMT) techniques for the treatment of non-fluent aphasia has been widely accepted by the rehabilitation medical community. However, consensus on which dimensions of speech function can be improved by NMT techniques and standardized intervention dosage remains elusive. This study aimed to provide evidence regarding the efficacy of NMT in improving speech function and explore the optimal intervention dose. A systematic review and meta-analysis were conducted to search for randomized clinical trials and open-label trials that evaluated speech functions after NMT.

**Methods:**

We searched all papers and reviews published from database inception to July 2023, including PubMed, Cochrane Library, Web of Science, Embase, and CNKI. Statistical analyses were mainly carried out on RevManV5.4.1 and pooled using a random-effects model. The primary outcome was the standardized mean difference (SMD) in speech functions, determined by calculating the change in speech functions score from baseline to the primary endpoint in the NMT group versus the control arm.

**Results:**

A total of 11 studies with 329 patients were included. NMT had a positive effect on repetition ability (SMD = 0.37, 95%CI [0.12, 0.62], *p*  < 0.05), but did not lead to significant differences in naming, comprehension, spontaneous speech, or communication. When the intervention time was >20 h, NMT exhibited a significant advantage at improving repetition ability (SMD = 0.43, 95%CI [0.06, 0.79], *p*  < 0.05).

**Discussion:**

This study provides evidence supporting the NMT enhancement of repetition ability in patients with non-fluent aphasia. Future large-sample studies are required to determine the optimal intervention dose of music therapy for different subtypes of non-fluent aphasia.

**Systematic review registration:**

PROSPERO, identifier CRD42023470313.

## 1 Introduction

Neurologic music therapy (NMT) as a developing branch of music therapy, uses the method of linking the plasticity of cerebral cortex with brain function to promote the rehabilitation of neurological disorders (1). The significant advances of clinical research in basic neuroscience have underscored the widespread recognition and rehabilitative potential of neurologic music therapy (NMT) for addressing speech dysfunctions caused by neurological disorders within the rehabilitation medical community. The NMT techniques commonly adopted for speech disorders include melodic intonation therapy (MIT), rhythmic speech cueing, voice pitch therapy, musical speech stimulation, and therapeutic singing (2). Among them, MIT has demonstrated efficacy as a music therapy technique for the treatment of broca’s aphasia in non-fluent aphasia (3). MIT involves simulating normal speech as a musical melody that resembles changes in intonation during speech, and patients are guided to articulate functional sentences through singing (4, 5). Moreover, therapeutic singing is widely applied to improve problems such as dysarthria, hypopnea, and speech fluency in speech disorders (6). When applying NMT to improve speech dysfunction, these techniques are often combined (2), enabling the synergistic use of melody, rhythm, tonality, harmony, and other musical elements to jointly activate music-processing areas in the right hemisphere, while also rewiring areas of speech control in the left hemisphere (7).

Aphasia is the most common sequela in stroke patients. About one-third of stroke survivors suffer from aphasia (8, 9), which persists in approximately 30–43% of these patients (10). Non-fluent aphasia is one of the most common types of aphasia, presenting as damage to the speech center in the left hemisphere (11). Patients with non-fluent aphasia retain the ability to sing despite their inability to utter meaningful sentences (4). As a result, they are encouraged to participate in music activities, which effectively enhances their motivation to engage in music therapy. Performing NMT in patients with non-fluent aphasia can enhance their respiratory function, articulation, and word finding and naming, while also improving their speech rhythm and prosody to some extent (12, 13).

Currently, several studies have confirmed the effectiveness of NMT for non-fluent aphasia; however, these investigations have not consistently reached a consensus regarding the specific dimensions of speech function that can be improved by NMT techniques. Li et al. (14) highlighted that MIT had a positive effect on language function, including spontaneous speech, comprehension, repetition, naming and reading skills, but not on writing skills. Zumbansen et al. (15) also found that choir singing could improve the comprehension and communication skills of patients with non-fluent aphasia, albeit without significant improvement in other dimensions of speech function. In addition, there is a lack of unified standards and evidence-based rationale for the intervention dose of NMT. Siponkoski et al. (16) suggested that the dose of music therapy was one session/week, 90 min/session, for a total of 24 h. However, Zhang et al. (17) revealed that the optimal intervention dose of MIT for patients with non-fluent aphasia was 30 min/session, 5 sessions/week, for a total of 20 h. Accordingly, we conducted extensive literature searches across five Chinese and English databases. Subsequently, we performed a meta-analysis to consolidate and analyze randomized controlled trials (RCTs) investigating the impact of NMT on the speech functions of patients with non-fluent aphasia. Specifically, we aimed to examine the efficacy of NMT techniques for improving various dimensions of speech function in patients with non-fluent aphasia, and determine the optimal intervention dose.

## 2 Methods

### 2.1 Search strategy and selection criteria

Literature searches were performed across five Chinese and English databases, namely PubMed, Cochrane Library, Web of Science, Embase, and CNKI. Owing to the insufficient number of studies, we expanded the search by using a combination of subject terms and free words. The search terms were as follows: “Speech Disorders or anomia* or aphasi*,” “music therapy or melodic intonation therapy or rhythmic speech cueing or vocal intonation therapy or sing*.” We conducted searches from database inception to July 2023, and further supplemented by reviewing the references and grey literature cited in the included studies. The details of the search strategy is described in Supplementary material 1.

Our inclusion criteria were as follows: (1) P: Adults aged 18 years and older with non-fluent aphasia; (2) I: NMT (e.g., MIT, rhythmic speech cueing, musical speech stimulation, therapeutic singing, and voice pitch therapy); (3) C: Other speech training or no training; and (4) O: Speech functions including naming, comprehension, repetition, spontaneous speech, and communication. We included RCT studies that followed the PICO format and were written in English or Chinese. Studies that combined music therapy with other interventions (such as drug therapy, artificial intelligence, transcranial magnetic stimulation, and transcranial direct current stimulation), those that solely involved listening to music as an intervention, those without outcome indicators related to speech functions, those without a full text and conference papers, were excluded.

### 2.2 Data collection and analysis

Four authors (JYG, WL, SQZ, CJL) were involved in screening the studies. Duplicates were simultaneously removed by the two authors (WL, CJL), and literature screening was independently performed by the other two authors (JYG, SQZ). Irrelevant studies were excluded after reading the study titles and abstracts, followed by full-text reading and review of potential studies for inclusion. Inconsistencies in study inclusion results were resolved by another author (XYZ).

Information, including country of publication, design type, sample size, disease type, aphasia type, intervention method, intervention dose, and outcome measures, were included in the table of study characteristics.

### 2.3 Quality assessment and bias identification

The quality of the included studies and risk of bias for each study were assessed according to the Cochrane Collaboration’s recommendations (18), which included selection, performance, detection, attrition, and reporting bias. Quality control and bias assessment were independently performed by the two authors (JYG, SQZ). In case of disagreements, consensus was reached through a discussion (Supplementary material 2).

### 2.4 Data and result extraction

Literature reading was independently performed by the two authors (JYG, SQZ) and data related to the study outcome indicators were extracted (Supplementary material 2). Another author (WL) checked and imported the data into the Review Manager V5.4.1 (Copenhagen: The Nordic Cochrane Centre, The Cochrane Collaboration, 2012) for meta-analysis.

### 2.5 Outcomes

Five outcome measures namely, naming, comprehension, repetition, spontaneous speech, and communication, were included in the meta-analysis. The assessment scales for the outcome measures included the Boston Diagnostic Aphasia Examination (BDAE), Amsterdam Nijmegen Everyday Language Test (ANELT), Western Aphasia Battery (WAB), Communicative Activity Log (CAL), and Chinese Rehabilitation Research Center Standard Aphasia Examinations (CRRCAE).

### 2.6 Statistical analysis

Statistical analyses were performed on the Review Manager V5.4.1 (RevMan V5.4.1) and Stata/MP 17.0. As different assessment tools were used; the effect size of measurement data was represented using the standardized mean difference (SMD). The SMD and 95% confidence interval (CI) of the pre- and post-intervention differences were employed to express the continuous variable outcomes. All data were subjected to pool analysis using a random-effects model. The heterogeneity of the included studies was tested based on the I^2^ values. An I^2^ value >50% and *p* < 0.1 indicated a high level of heterogeneity, and further subgroup analysis was required to determine the source of heterogeneity. Sensitivity analysis was performed on each subgroup using the leave-one-out method. The significance level for meta-analysis was *α* = 0.05. Publication bias was analyzed using funnel plot. A forest plot analysis was performed by pooling the same outcome indicators, and sensitivity analysis was performed on the outcome measures for each group.

## 3 Results

### 3.1 Study selection

**Figure 1:**
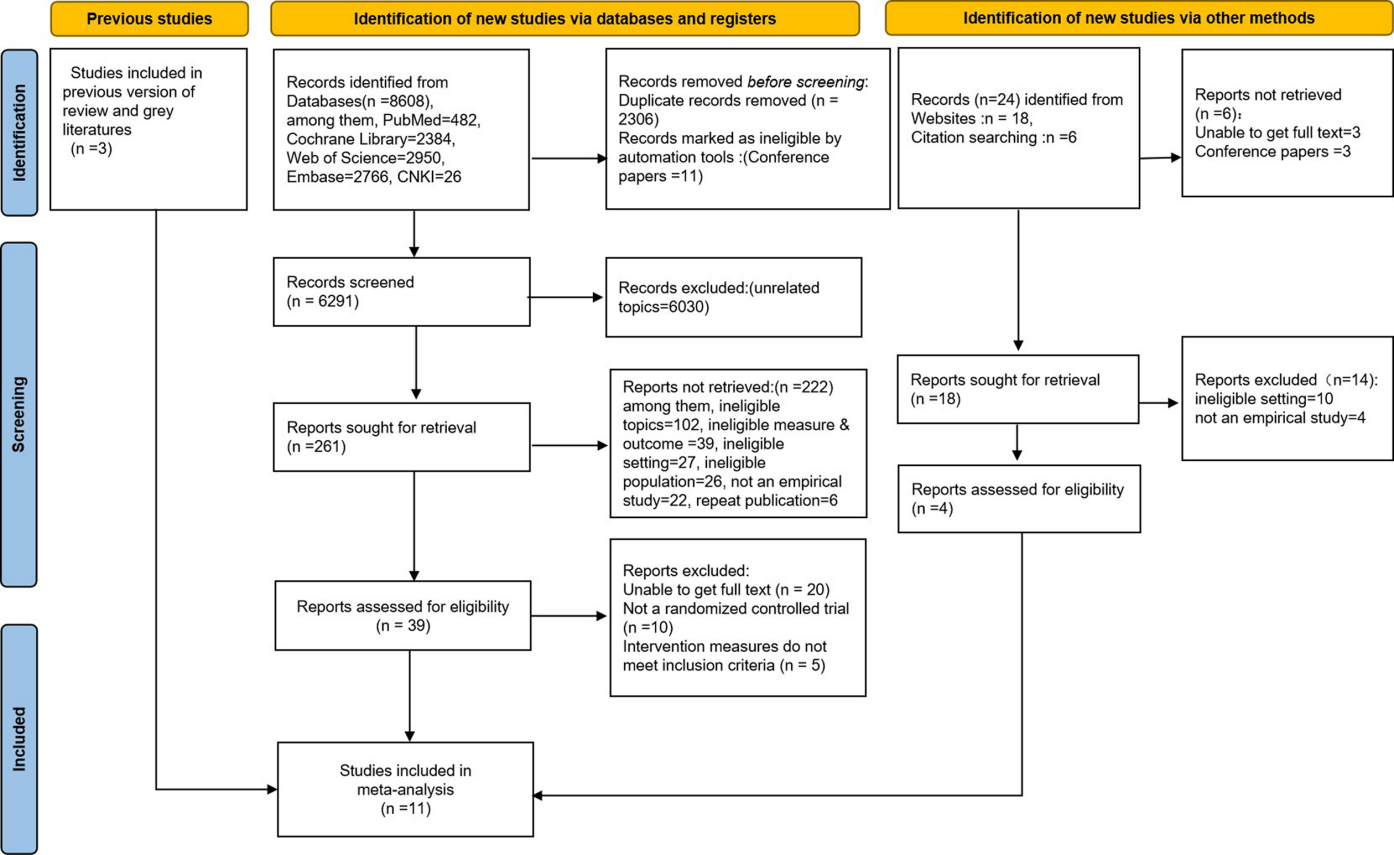
PRISMA flow diagram of the selection procedure.

A total of 8,635 studies were retrieved, and the studies were supplemented by checking the citations. After checking for duplicates, reading the abstracts, and subsequently the full text for precise screening, a total of 11 studies were finally included. The literature screening process is shown in Figure 1. The list of studies that were excluded after refined screening is provided in Supplementary material 3.

### 3.2 Study characteristics

The eleven studies (14–17, 19–25) comprise 329 patients, with 167 and 162 patients in the experimental (EG) and control group (CG), respectively. The between-group comparisons of general information across all included studies did not reveal any significant differences. Regarding outcome measures, seven studies assessed the patients’ naming, nine assessed their comprehension, ten assessed their repetition, five assessed their spontaneous speech, and three assessed their communication. The basic characteristics of the included studies are provide in Table 1.

**Table 1:** Characteristics of the included studies (*N* = 11).

Source	Overall sample size	Research site	Study design	Group sample size	Lesion etiology	Age of samples (M ± SD, year)	Types of speech disorders	Intervention	Intervention Dose	Time point for evaluation	Outcomes
Conklyn et al. (19)	24	USA	RCT, parallel-group design	EG:14 CG:10	Stroke	EG: 56.8 ± 17.11 CG: 66.9 ± 11.77	Non-fluent aphasia	EG: Modified melodic intonation therapy (MMIT) CG: No treatment	10- to 15-min/session; 3 sessions (0.75 h)	Prior to each session and the post-test immediately after the session	②③
Van Der Meulen et al. (24)	24	The Netherlands	RCT, cross-over design	EG:13 CG:11	Stroke	EG: 53.1 ± 12.0 CG: 52.0 ± 6.6	Subacute aphasia	EG: MIT (6 weeks) + without therapy (6 weeks) CG: without therapy (6 weeks) + MIT (6 weeks)	(5 h/w) during 6 weeks (30 h)	Baseline, 6 weeks later later, 12 weeks later	①③
Zumbansen et al. (15)	14	Canada	RCT, parallel-group design	EG:7 CG:7	Stroke and brain tumor	EG: 63.4 ± 7.5 CG: 54.0 ± 11.6	Chronic aphasia	EG: Choir sessions CG: Waiting list	Weekly 2-h long; 26 weeks (52 h)	Baseline, 6 months later	①②③⑤
Raglio et al. (21)	20	Milan	RCT, parallel-group design	EG:10 CG:10	Stroke	EG: from 42 year to 89 year CG: from 42 to 89 year	Aphasia	EG: Music Therapy (MT) + Speech and Language Therapy (SLT) CG: Speech and Language Therapy	EG: 30 min/session, twice a week for 15 weeks, with a total of 30 sessions (22.5 h) CG: 45 min/session, twice a week for 15 weeks, with a total of 30 sessions	Baseline, 15 weeks later	①④
Van Der Meulen et al. (23)	17	The Netherlands	RCT, cross-over design	EG:10 CG:7	Stroke	EG: 58.1 ± 15.2 CG: 63.6 ± 12.7	Chronic aphasia	EG: MIT (6 weeks) + without therapy (6 weeks) CG: without therapy (6 weeks) + MIT (6 weeks)	Face-to-face therapy time was 3 h/week; 6 weeks (18 h) Used the iPod to practice at home, at least 2 h/week, but no more than 7 h/week	Baseline, 6 weeks later, 12 weeks later	①②③
Haro-Martínez et al. (20)	20	Spain	RCT, cross-over design	EG:10 Group CG:10	Stroke	EG: from 38 year to 81 year CG: from 38 year to 81 year	Non-fluent aphasia	EG: MIT (6 weeks) + without therapy (6 weeks) CG: without therapy (6 weeks) + MIT (6 weeks)	30 min/session; (12 sessions over 6 weeks); (6 h)	Baseline, 6 weeks later,12 weeks later	②③⑤
Sun et al. (22)	40	China	RCT, parallel-group design	EG:20 CG:20	Stroke	EG: 52.20 ± 15.98 CG: 52.00 ± 12.10	Broca’s aphasia	EG: MIT CG: Schuell’s stimulation therapy	30 min each session, 5 days a week for 60 sessions (30 h)	Baseline, 12 weeks later	①②③④
Li et al. (14)	40	China	RCT, parallel-group design	EG:20 CG:20	Stroke	EG: 64.3 ± 2.4 CG: 66.5 ± 2.6	Broca’s aphasia	EG: MIT CG: Speech and Language Therapy (SLT)	1.5 h each session, 5 days a week for 4 weeks (30 h)	Baseline, 4 weeks later	②③
Zhang et al. (17)	40	China	RCT, parallel-group design	EG:20 CG:20	Stroke	EG: From 18 year to 70 year CG: From 18 year to 70 year	Non-fluent aphasia	EG: MIT training CG: Speech therapy	30 min/day, five times a week for 8 weeks (20 h)	Baseline, 8 weeks later	①②③④
Siponkoski (16)	50	Finland	RCT, cross-over design	EG:23 CG:27	Cerebrovascular accident Traumatic brain injury	EG:63.5 ± 10.3 CG:64.0 ± 12.3	Aphasia	A: Group training: Singing +MIT B: Home training EG: AB CG:BA	A:1 session/week, 1.5 h/session, Group training sessions comprised 60 min of singing training for PWAs and FCs and 30 min of group-based MIT for PWAs; total 24 h B: 3 sessions/week, 30 min/session, 16 weeks, total 24 h.	Baseline, 5 months later, 9 months later	②④⑤
Zhang et al. (25)	40	China	RCT, parallel-group design	EG:20 CG:20	Stroke	EG:50.15 ± 15.44 CG:51.6 ± 14.27	Non-fluent aphasia	EG: MIT CG: Speech and Language Therapy (SLT)	30 min each session, 5 days a week for 4 weeks (10 h)	Baseline, 4 weeks later	①②③④

EG, Experimental Group; CG, Control Group; M, Mean; SD, Standard deviation. ①Naming, ②Comprehension, ③Repetition, ④Spontaneous speech, ⑤Communication.

### 3.3 Literature quality evaluation

**Figure 2:**
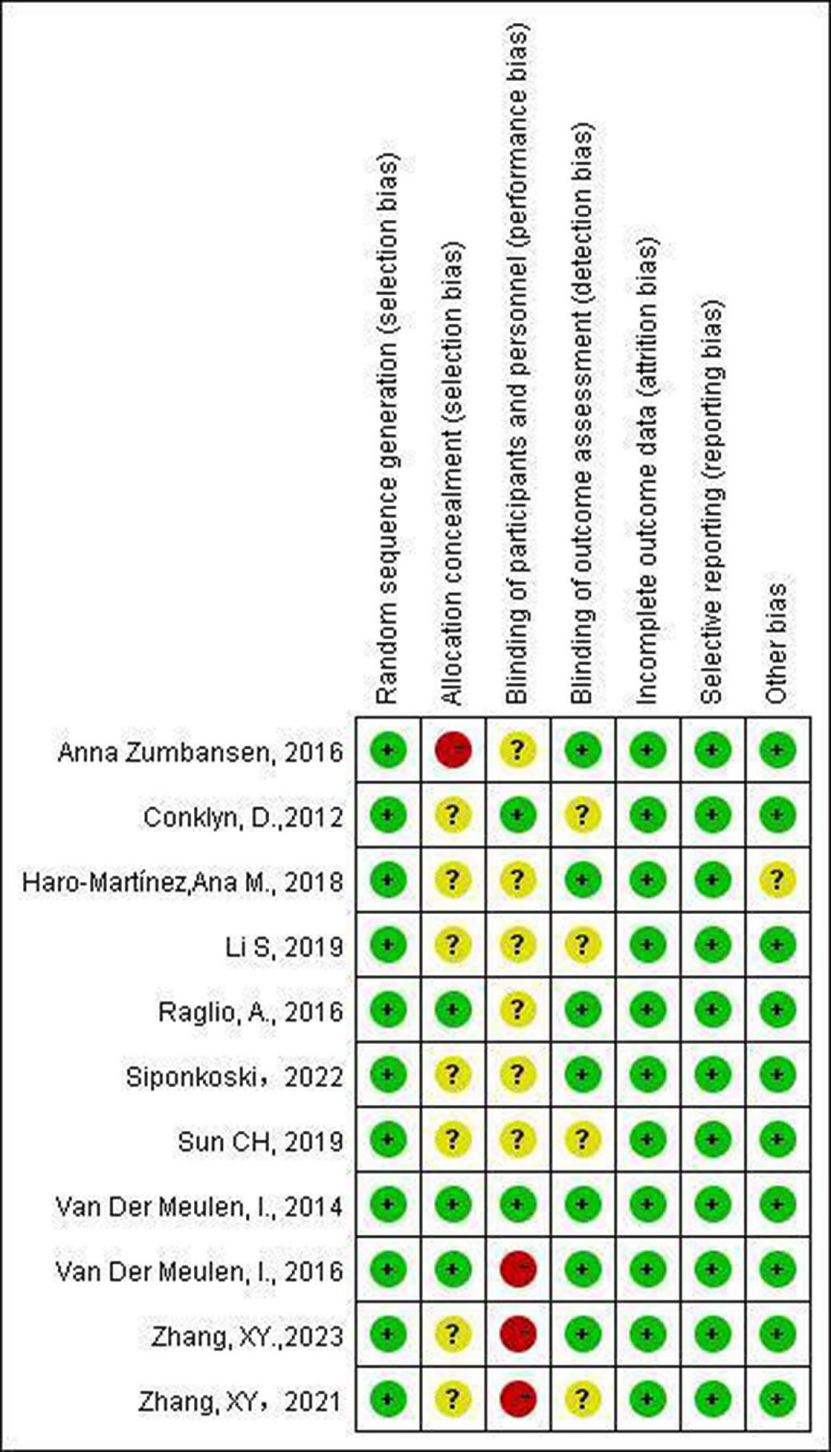
The risk of bias in included studies.

The quality of the 11 included studies was assessed using the Cochrane Handbook (26) and the results are shown in Figure 2. All studies provided clear description of the generation of random sequences. Three studies (21, 23, 24) explicitly described the methods for allocation concealment. All studies explicitly reported comparability at baseline between groups. Performance bias was marked as uncertain in most studies due to the specificity of the intervention, making it impossible to completely blind the investigators and participants. Most studies followed the design principle of blinding the assessors. All studies presented complete outcome data, with no selective reporting.

### 3.4 Effectiveness of NMT on level of naming

**Figure 3:**
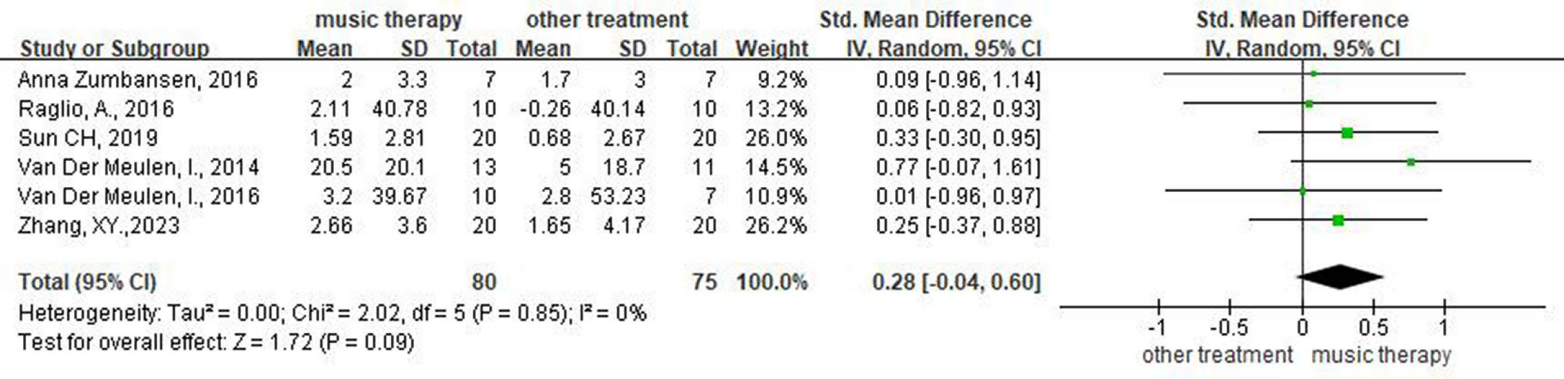
Forest plot of the effect of NMT on naming.

Six studies (15, 21–25) assessed patients’ naming ability. A meta-analysis using a random-effects model showed that the difference between the two groups was not statistically significant (SMD = 0.28, 95%CI [−0.04, 0.60], *p* > 0.05), suggesting that the EG did not differ from the CG with respect to enhancing patients’ naming ability (Figure 3).

### 3.5 Effectiveness of NMT on level of comprehension

**Figure 4:**
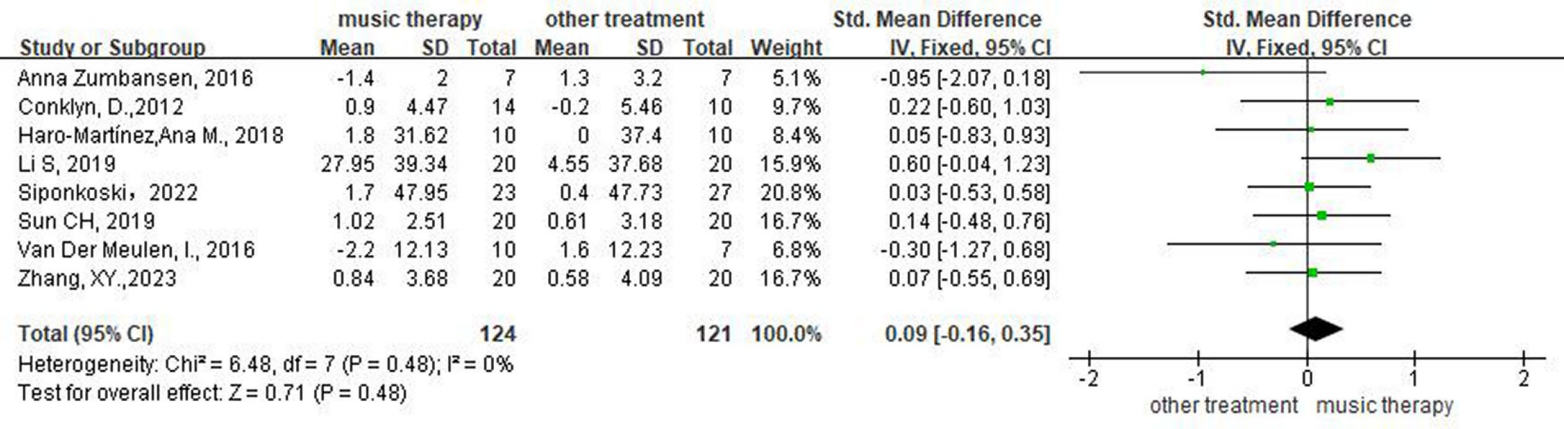
Forest plot of the effect of NMT on comprehension.

Eight studies (14–16, 19, 20, 22, 23, 25) assessed the patients’ comprehension. Meta-analysis using a random-effects model demonstrated that the difference between the two groups was not statistically significant (SMD = 0.09, 95%CI [−0.16, 0.35], *p* > 0.05), indicating that the EG did not differ from CG with respect to enhancing patients’ comprehension (Figure 4).

### 3.6 Effectiveness of NMT on level of repetition

**Figure 5:**
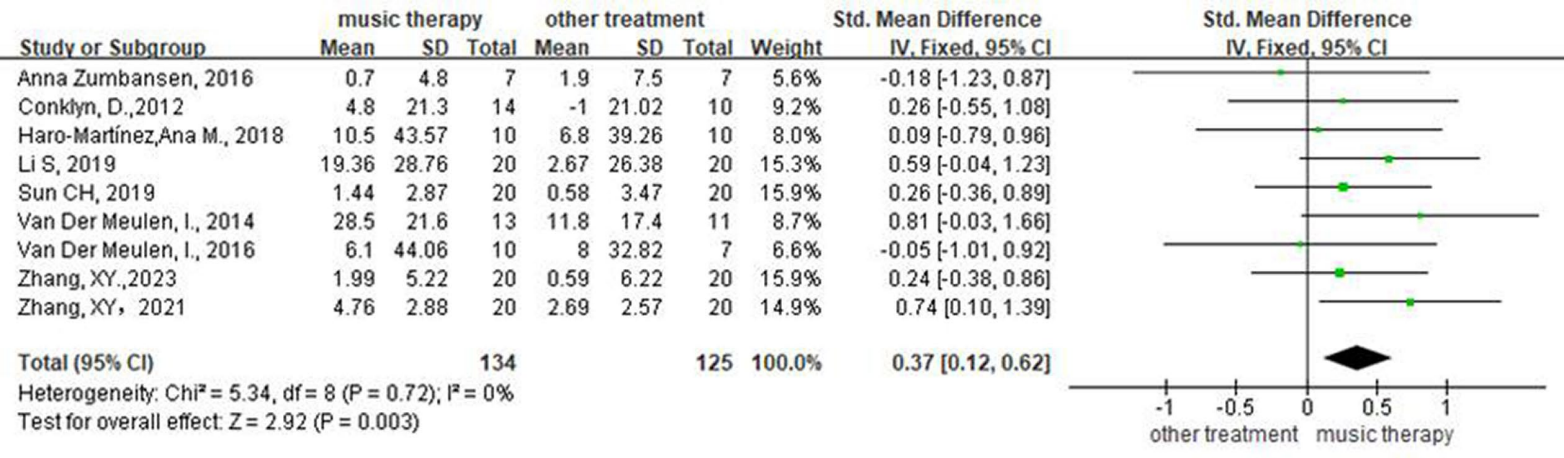
Forest plot of the effect of NMT on repetition.

Nine studies (14, 15, 17, 19, 20, 22–25) assessed patients’ repetition. Meta-analysis using a random-effects model showed that the difference between the two groups was statistically significant (SMD = 0.37, 95%CI [0.12, 0.62], *p* < 0.05), indicating that the EG was superior to CG in enhancing patients’ repetition (Figure 5).

### 3.7 Effectiveness of NMT on level of spontaneous speech

**Figure 6:**
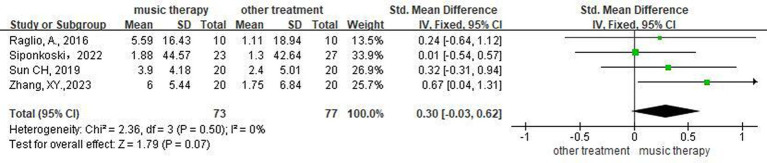
Forest plot of the effect of NMT on spontaneous speech.

Four studies (16, 21, 22, 25) assessed patients’ spontaneous speech. Meta-analysis using a random-effects model showed that the difference between the two groups was not statistically significant (SMD = 0.30, 95%CI [−0.03, 0.62], *p* > 0.05), suggesting that the EG did not differ from the CG with respect to enhancing the patients’ spontaneous speech (Figure 6).

### 3.8 Effectiveness of NMT on level of communication

**Figure 7:**
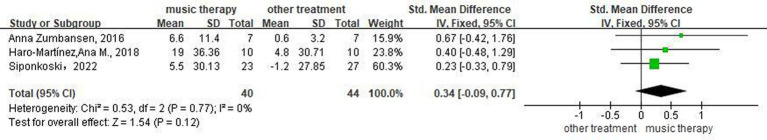
Forest plot of the effect of NMT on communication.

Three studies (15, 16, 20) assessed patients’ communication. A meta-analysis using a random-effects model showed that the difference between the two groups was not statistically significant (SMD = 0.34, 95%CI [−0.09, 0.77], *p* > 0.05), suggesting that the EG did not differ from CG with respect to enhancing patients’ communication (Figure 7).

### 3.9 Intervention dose

**Figure 8:**
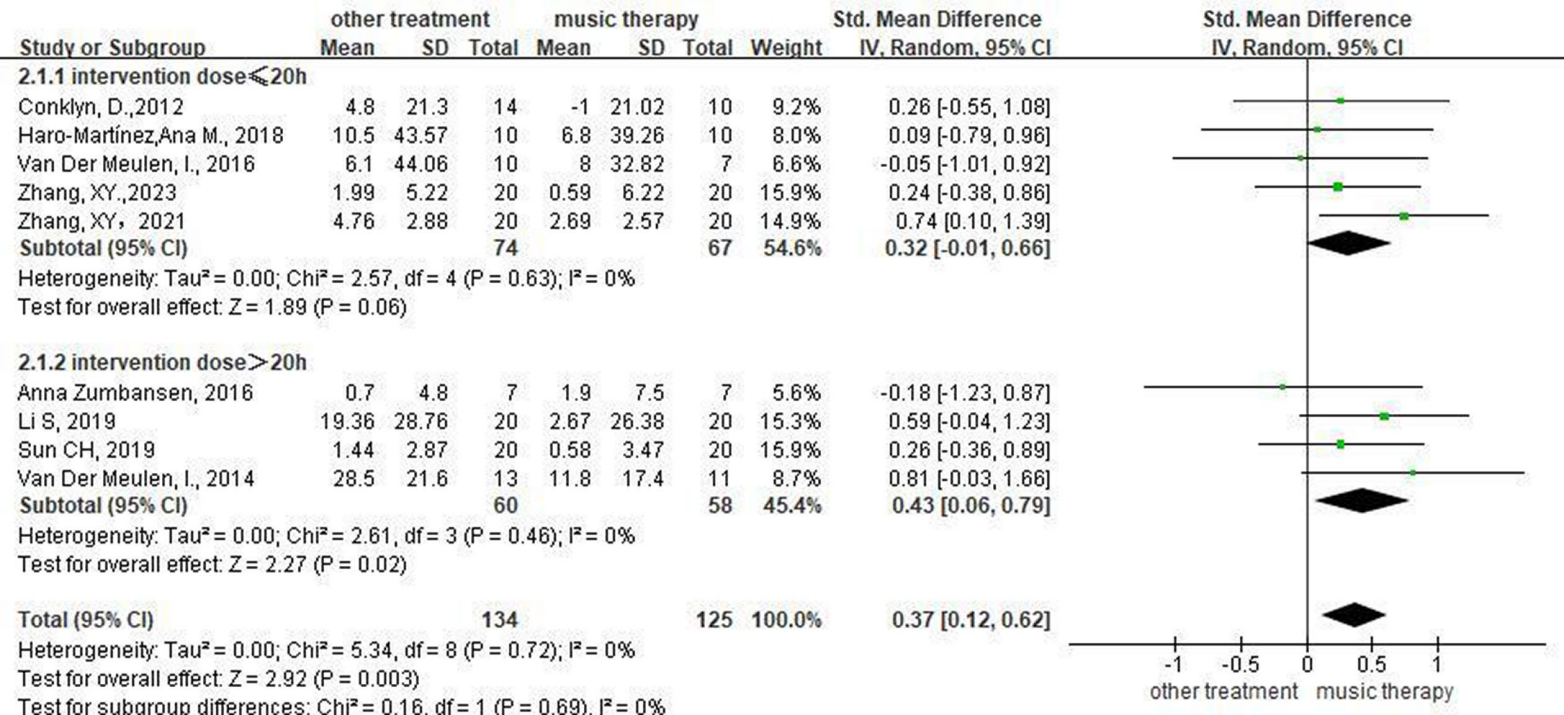
Forest plot of the effect of NMT on repetition for subgroup analysis based on intervention dose.

Exploratory grouping of intervention doses was performed on the included studies that examined repetition (Supplementary material 4). Meta-integration of any study with an intervention dose of 30 h and ≤ 20 h was performed using a random-effects model, which all resulted in statistically significant differences between the music therapy group and other intervention methods. Meta-integration using a random-effects model of studies with intervention doses ≤30 h but not ≤20 h, yielded statistically significant differences between the music therapy group and other intervention methods (Supplementary material 4). Therefore, an intervention dose of 20 h was selected as the cut-off for subgroup analysis to compare the effect of NMT on the repetition of patients receiving different intervention doses, and a meta-analysis was performed using a random-effects model. Five studies (17, 19, 20, 23, 25) with NMT intervention doses ≤20ours h showed no significant difference between the two groups (SMD = 0.32, 95%CI [−0.01, 0.66], *p* > 0.05), indicating that the EG did not differ from the CG with respect to enhancing patients’ repetition. Four studies (14, 15, 22, 24) with NMT intervention doses >20 h showed a statistically significant difference between the two groups (SMD = 0.43, 95%CI [0.06, 0.79], *p* < 0.05), indicating that the EG was superior to the CG in enhancing patients’ repetition (Figure 8).

### 3.10 Heterogeneity and sensitivity analysis

The I^2^ of all studies was >50% (*p* > 0.05), indicating no significant heterogeneity among the pooled studies and no further subgroup analysis was required. Stata/MP 17.0 for Windows (Copenhagen: Computer Resource Center, 2021) was used to conduct sensitivity analysis on the outcome measures using the leave-one-out method. The results showed that for repetition, after any one study was excluded, the pooled results of the remaining studies remained statistically significant (95%CI not including 0). This was consistent with the original pooled results, indicating that the results were stable. For naming, comprehension, and communication, after any one study was excluded, the pooled results of the remaining studies remained statistically insignificant (95%CI including 0). This finding was consistent with the original pooled results, indicating that the results were stable. For spontaneous speech, after any one of three out of four included studies was excluded, the pooled results of the remaining studies were not statistically significant (95% CI including 0), which was consistent with the original pooled results, indicating that the results were stable (Supplementary material 5).

### 3.11 Certainty of evidence

**Figure 9:**
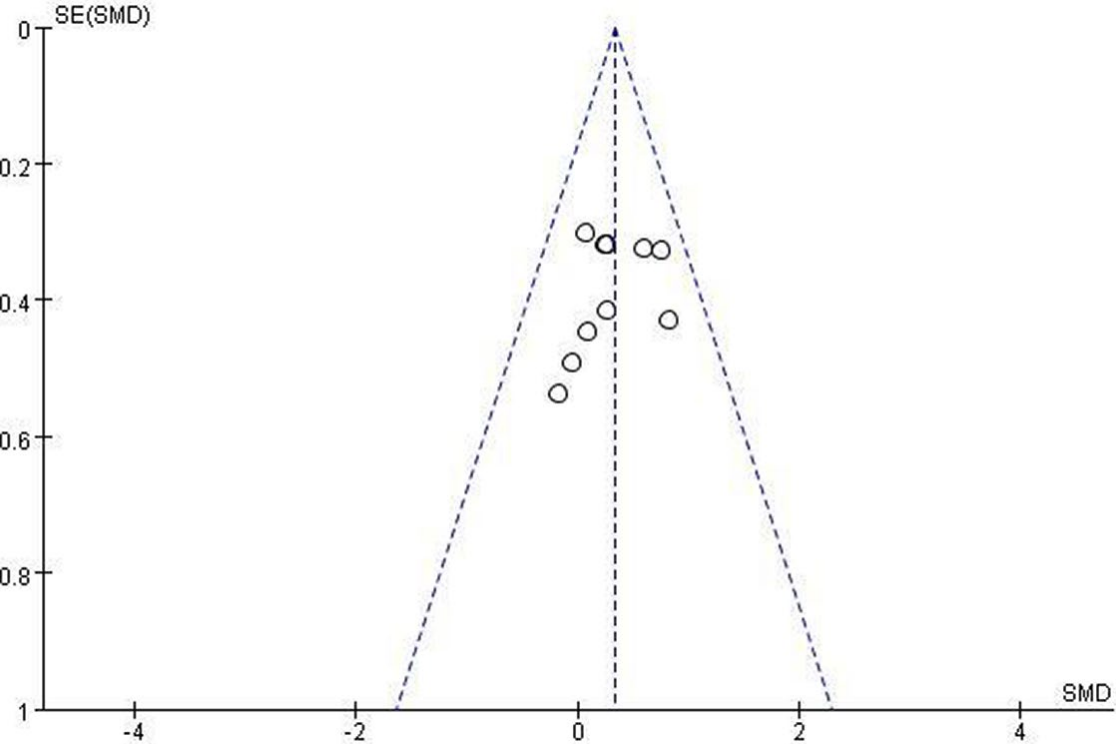
Funnel diagram constructed with repetition.

Using the rating method recommended by the GRADE system (27), the quality of evidence was assessed based on five domains: risk of bias, inconsistency, indirectness, imprecision, and publication bias. The results showed that the methodological quality of the included literature was moderate for naming, comprehension, repetition, and communication, and high for spontaneous speech (Table 2). The Cochrane Risk of Bias Assessment Tool was used to assess the limitations of the included RCTs. When the total weight ratio of high-risk literature was >20% due to irrational design or unclear descriptions of randomization, allocation concealment, blinding (e.g., implementation and measurement), then downgrading should be considered. Therefore, naming, comprehension, and repetition were each downgraded by one level for risk of bias. The pooled I^2^ for all outcomes was >50% (*p* > 0.05), and the differences in the intervention types, doses, and durations among the included studies were small; therefore, no study results were downgraded for heterogeneity. The patient populations, interventions, and important outcome indicators were largely consistent across all included RCTs, and hence no study results were downgraded for indirectness. In terms of imprecision, the quality of evidence for communication was downgraded due to wide CI or limited sample size. Publication bias was visually evaluated using funnel plot. Repetition, which had the largest number of included studies, was selected as the main outcome to create the funnel plot. Our findings revealed that most studies had large sample sizes and were concentrated in the narrow, upper part of the funnel plot, suggesting that the result was relatively robust. Furthermore, Egger’s test yielded *p* = 0.251, and thus the result was not downgraded. The funnel plot is shown in Figure 9.

**Table 2:** Grade evaluation of evidence quality.

Certainty assessment							No of patients		Effect	Certainty	Importance
№ of studies	Study design	Risk of bias	Inconsistency	Indirectness	Imprecision	publication bias	Music therapy	placebo	Absolute (95% CI)		
Effects of NMT on naming function											
6	Randomized trials	Serious^a^	Not serious	Not serious	Not serious	Not serious	80	75	SMD 0.28 SD more (−0.04 fewer to 0.60 more)	⨁⨁⨁◯ Moderate	9-Critical
Effects of NMT on comprehension function											
8	Randomized trials	Serious^a^	Not serious	Not serious	Not serious	Not serious	124	121	SMD 0.09 SD more (−0.16 fewer to 0.35 more)	⨁⨁⨁◯ Moderate	9-Critical
Effects of NMT on repetition function											
9	Randomized trials	Serious^a^	Not serious	Not serious	Not serious	Not serious	134	125	SMD 0.37 SD more (0.12 more to 0.62 more)	⨁⨁⨁◯ Moderate	9-Critical
Effects of NMT on spontaneous speech function											
4	Randomized trials	Not serious	Not serious	Not serious	Not serious	Not serious	73	77	SMD 0.30 SD more (−0.03 fewer to 0.62 more)	⨁⨁⨁⨁ High	9-Critical
Effects of NMT on communication function											
3	Randomized trials	Not serious	Not serious	Not serious	Not serious	Not serious	40	44	SMD 0.34 SD more (−0.09 fewer to 0.77 more)	⨁⨁⨁◯ Moderate	9-Critical

CI, confidence interval; SMD, standardized mean difference. ^a^According to the Cochrane risk assessment criteria, the weight of the literature with high-risk items is more than 20%.

## 4 Discussion

Based on previously published systematic reviews and meta-analyses concerning the improvement of non-fluent aphasia through MIT (28), we further expanded the intervention methods in music therapy, and included studies that adopted music therapy modalities such as: choir singing (15), singing (16), MIT (14, 16, 17, 19, 20, 22–25) and other modalities (free sound-music improvisation) (21). Our meta-analysis showed that only NMT significantly improved the repetition ability of patients with non-fluent aphasia (*p* < 0.05), which is consistent with the results of an RCT conducted by Van Der Meulen et al. (23). This may be due to the fact that the music therapy modalities adopted in the included studies were mainly MIT and singing. In the speech practice sessions of these intervention methods, the pitch, melody, and lyrics are formulaic or fixed (29), whereas naming, comprehension, spontaneous speech, and communication skills require better training in a more flexible language environment (23, 30). However, this finding does not negate the importance of MIT and singing in improving the speech function of patients with non-fluent aphasia. Repetition and imitation are the basis of language learning (31), while the training of comprehension, spontaneous speech, and communication are all predicated upon good repetition abilities. In addition, these results imply that after MIT or singing has elevated the patient’s repetition ability to a relatively advanced level, integrating additional, more elaborate richer music therapy modalities becomes necessary to augment other aspects of speech function. Modalities such as musical speech stimulation, lyrics adaptation, and musical improvisation, could contribute to enhancing naming ability, comprehension, spontaneous speech, and communication (32–34).

The intervention dose of music therapy has been rarely explored. One study (25) provided recommendations for the therapeutic dose of MIT for different subtypes of non-fluent aphasia based on clinical experience; however, no RCTs or evidence-based studies have verified these recommendations. Thus, we conducted a preliminary analysis and explored the intervention doses of the nine included studies that examined repetition as an outcome. Our findings revealed that music therapy was superior to other interventions when the intervention dose was >20 h. However, in actual small-sample clinical trials (20, 25), patients’ repetition ability significantly improved when the total duration of NMT was close to 10 h. This may be due to the inconsistent treatment effects caused by the proficiency of the music therapy practitioner in clinical work, differences in treatment protocols, and varying subtypes of non-fluent aphasia. Due to the small number of included studies, the subtypes of non-fluent aphasia presented by the included participants were not specified. Therefore, our findings cannot yet be regarded as conclusive evidence for determining the optimal intervention dose, and can only serve as a reference for designing intervention duration in future studies. Further analysis and validation of the optimal intervention dose for various subtypes of non-fluent aphasia will be necessary through large-sample studies.

This study has some limitations. First, a limited number of studies were included (*n* = 11), the included studies had small sample sizes, and the follow-up time was relatively short, which may lead to insufficient evidence. Second, triple-blinding is difficult to implement for this type of RCTs. Although the quality of included RCTs was high, there was still some bias, and none of the studies fully demonstrated the effect of music therapy on repetition, resulting in a moderate level of evidence for this outcome measure.

## 5 Conclusion

This study provides evidence supporting the NMT enhancement of repetition in patients with non-fluent aphasia. Future large-sample studies are required to determine the optimal intervention dose of music therapy for different subtypes of non-fluent aphasia.

## Data availability statement

The original contributions presented in the study are included in the article/Supplementary material, further inquiries can be directed to the corresponding authors.

## Author contributions

JG: Writing – original draft, Writing – review & editing, Data curation, Formal analysis, Methodology, Resources. WL: Writing – original draft, Writing – review & editing, Data curation, Formal analysis, Resources. SZ: Writing – original draft, Writing – review & editing, Data curation, Resources. CL: Writing – original draft, Writing – review & editing, Data curation, Resources. CF: Writing – review & editing, Funding acquisition, Supervision, Validation. XZ: Funding acquisition, Validation, Writing – review & editing, Methodology, Supervision.
