# Supplementary material for: Neurologic music therapy for non-fluent aphasia: a systematic review and meta-analysis of randomized controlled trials
Source: Front Neurol. 2024 May 23;15:1395312. doi: 10.3389/fneur.2024.1395312 (PMC11153767; doi:10.3389/fneur.2024.1395312)

**Supplementary material 1 Search strategy**

| PubMed | 1# Search: Speech Disorders or language disorders or anomia[MeSH Terms] 99760  2# Search: aphasi* or dysphasi* or dysarthria[MeSH Terms] 25046  3# Search: (speech[Title/Abstract] OR language*[Title/Abstract] OR linguistic[Title/Abstract] OR communicate*[Title/Abstract]) AND (disorder*[Title/Abstract] OR impair*[Title/Abstract] OR problem*[Title/Abstract] OR dysfunction[Title/Abstract] OR difficult*[Title/Abstract]) 105359  4# 1# OR 2# OR 3# 168117  5# Search: (Music Therapy or melodic intonation therapy or rhythmic speech cueing or vocal intonation therapy[MeSH Terms]) OR (musical speech stimulation or therapeutic singing[MeSH Terms]) 9851  6# Search: sing* or song* or chant* or compose or composing or improvise* or chord* or harmony* or melody* or rhythm* 3195358  7# 5# OR 6# 3202790  8# 4# AND 7# 16818  9# Filters Randomized Controlled Trial 482 |
| --- | --- |
| Cochrane Library | 1# Search: Speech Disorders or language disorders or anomia[MeSH Terms] 1930  2# Search: aphasia* or dysphasia* or Dysarthria [MeSH Terms] 2783  3# Search: (speech[Title/Abstract] OR language*[Title/Abstract] OR linguistic[Title/Abstract] OR communicate*[Title/Abstract]) AND (disorder*[Title/Abstract] OR impair*[Title/Abstract] OR problem*[Title/Abstract] OR dysfunction[Title/Abstract] OR difficult*[Title/Abstract]) 17950  4# 1# OR 2# OR 3# 20424  5# Search: (Music Therapy or melodic intonation therapy or rhythmic speech cueing or vocal intonation therapy[MeSH Terms]) OR (musical speech stimulation or therapeutic singing[MeSH Terms]) 4109  6# Search: sing* or song* or chant* or compose or composing or improvise* or chord* or harmony* or melody* or rhythm* 267408  7# 5# OR 6# 270644  8# 4# AND 7# 4018  9# Filters Randomized Controlled Trial 2384 |
| Web of Science | 1# Search: TS=( Speech Disorders OR anomia OR language disorders OR dysarthria OR aphasi* OR dysphasi*) 198469  2# Search: TS=(speech OR language* OR linguistic OR communicate*) AND (disorder* OR impair* OR problem* OR dysfunction OR difficult*) 866161  3# 1# OR 2# 896393  4# Search: TS=( Music Therapy OR musical speech stimulation OR melodic intonation therapy OR rhythmic speech cueing OR vocal intonation therapy OR therapeutic singing) 18469  5# Search:TS=(sing* OR song* OR chant* OR compose OR chord* OR harmony* OR melody* OR composing OR improvise* OR rhythm*) [7](https://www.webofscience.com/wos/alldb/summary/93f051d9-0c81-4f60-ade0-c69d881cb5bf-96fc4274/relevance/1)991521  6# 4# OR 5# OR 7# 8005725  7# 3# AND 6# 69520  8# Search: TS=(randomized controlledtrial OR randomized OR placebo OR random OR randomised) 2572535  9# 7# AND 8# 2950 |
| Embase | 1# Search: Speech Disorders or language disorders or anomia or dysarthria or aphasia or dysphasia [Emtre thesaurus] 158973  2# Search: ('speech':ab,kw,ti OR 'language*':ab,kw,ti OR 'linguistic':ab,kw,ti OR 'communicate*':ab,kw,ti) AND ('disorder*':ab,kw,ti OR 'impair*':ab,kw,ti OR 'problem*':ab,kw,ti OR 'dysfunction':ab,kw,ti OR 'difficult*':ab,kw,ti) 145663  3# 1# OR 2# 266385  4# Search: Music Therapy [Emtre thesaurus] 9629  5# Search:'musical speech stimulation':ab,kw,ti OR 'melodic intonation therapy':ab,kw,ti OR 'rhythmic speech cueing':ab,kw,ti OR 'vocal intonation therapy':ab,kw,ti OR 'therapeutic singing':ab,kw,ti 131  6# Search:'sing*':ab,kw,ti OR 'song*':ab,kw,ti OR 'chant*':ab,kw,ti OR 'compose':ab,kw,ti OR 'chord*':ab,kw,ti OR 'harmony*':ab,kw,ti OR 'melody*':ab,kw,ti OR 'composing':ab,kw,ti OR 'improvise*':ab,kw,ti OR 'rhythm*':ab,kw,ti 2932104  7# 4# OR 5# OR 7# 2940179  8# 3# AND 7# 21089  9# Search: 'crossover procedure':de OR 'double-blind procedure':de OR 'randomized controlled trial':de OR 'single-blind procedure':de OR (random* OR factorial* OR crossover* OR cross NEXT/1 over* OR placebo* OR doubl* NEAR/1 blind* OR singl* NEAR/1 blind* OR assign* OR allocat* OR volunteer*):de,ab,ti 3161285  10# 8# AND 9# 2766 |
| CNKI | (音乐治疗 + 神经音乐治疗 + 旋律发音治疗 + 节奏性语言提示 + 音乐语言刺激 + 治疗性歌唱 + 口腔呼吸运动训练 + 音乐发育性言语训练 + 声音音调治疗 + 音乐象征性交流训练)AND(构音障碍 + 失语症 + 口吃 + 言语障碍) 26 |

PS: As of July 12, 2023


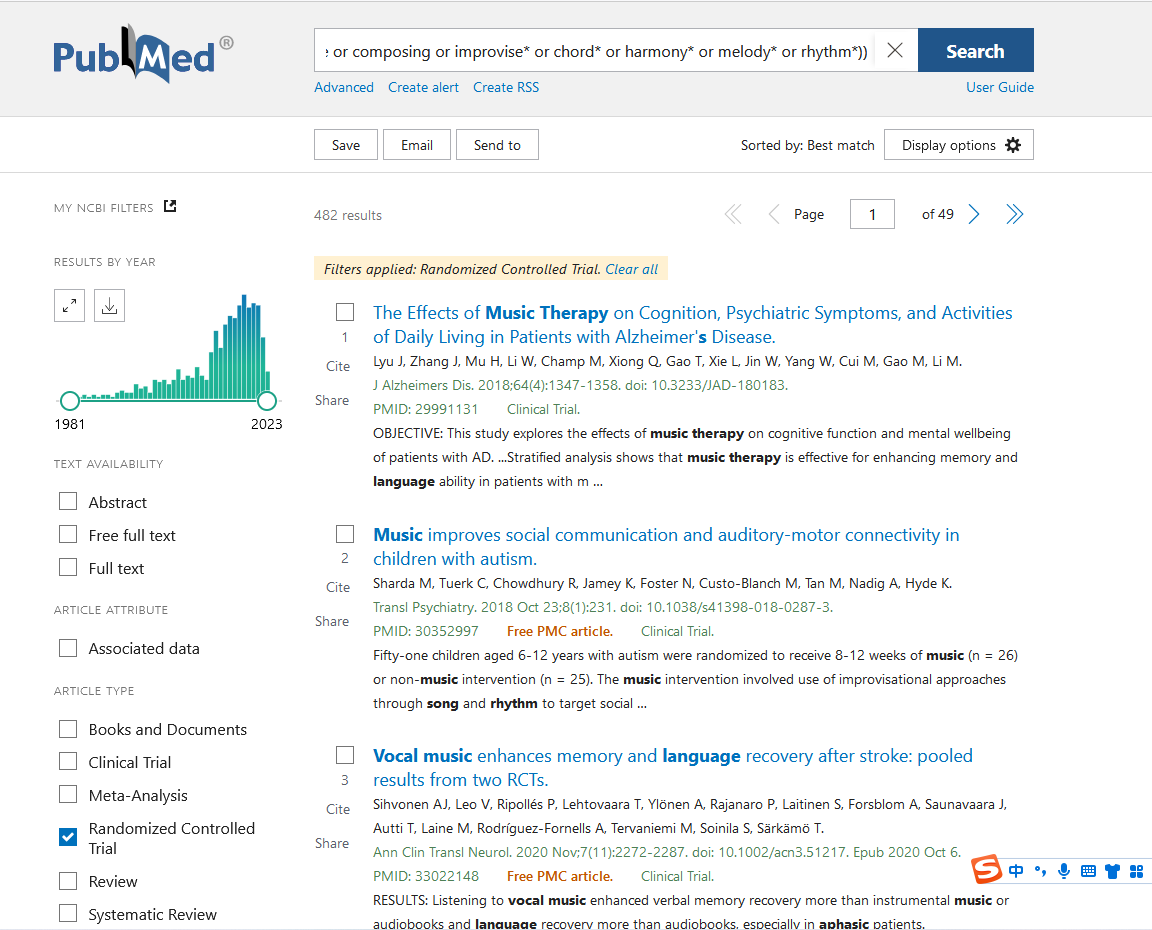


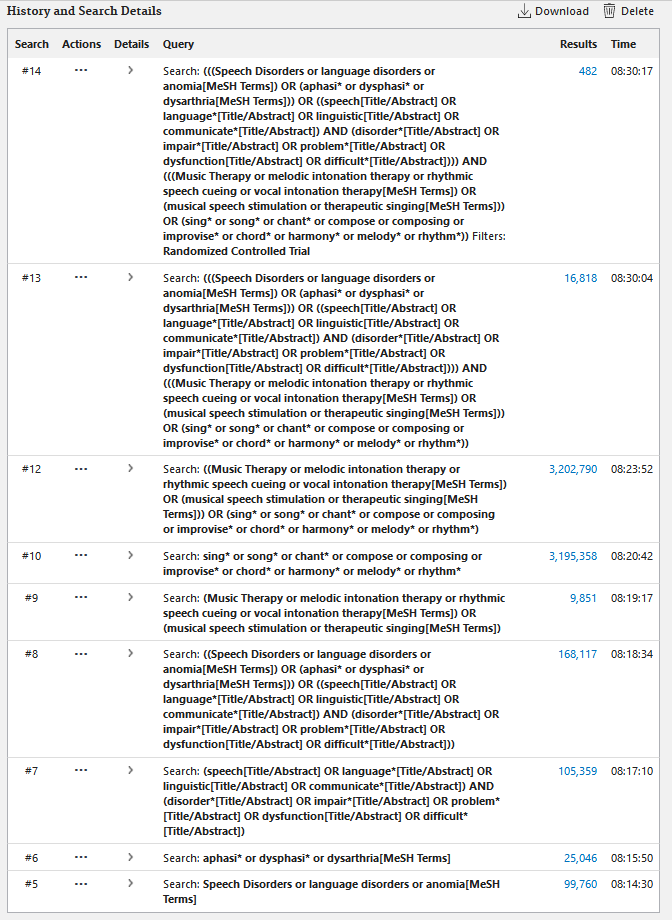

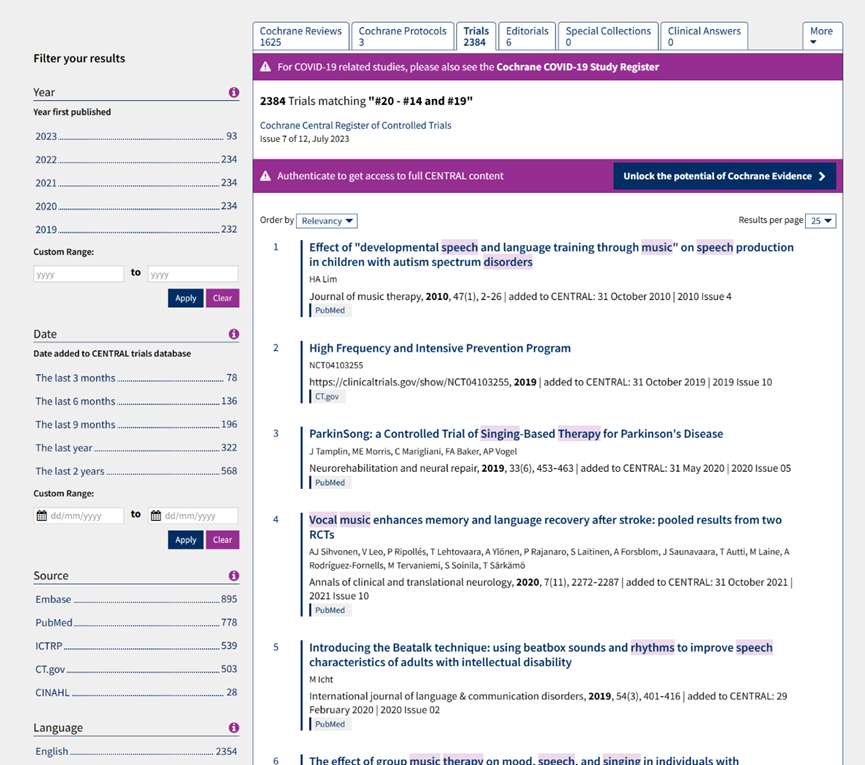


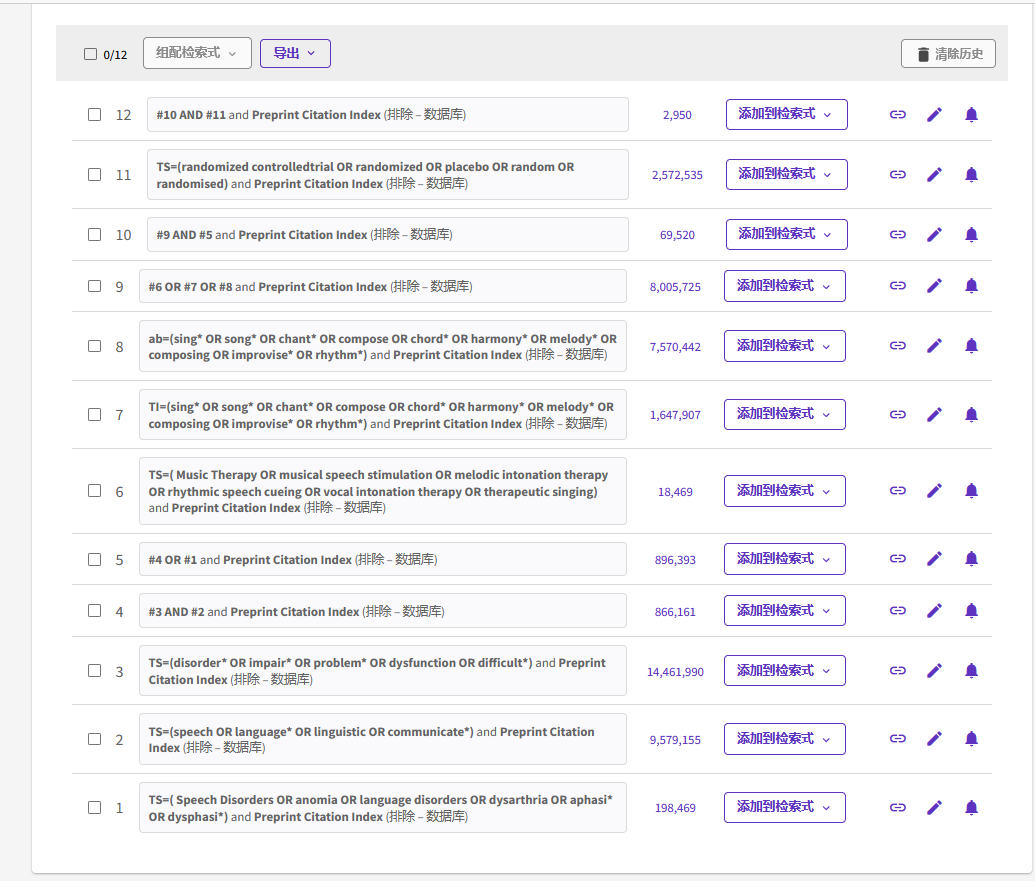


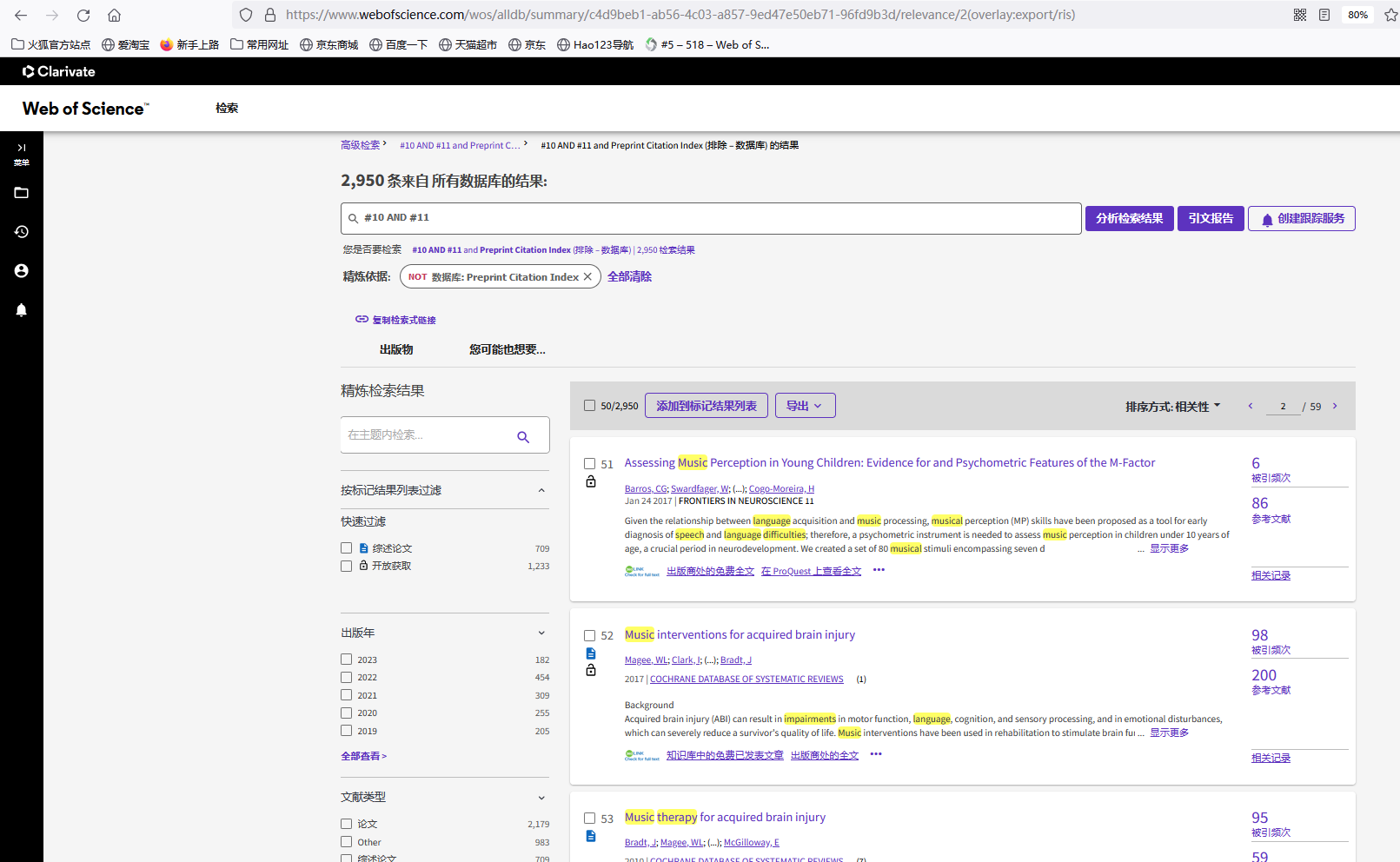

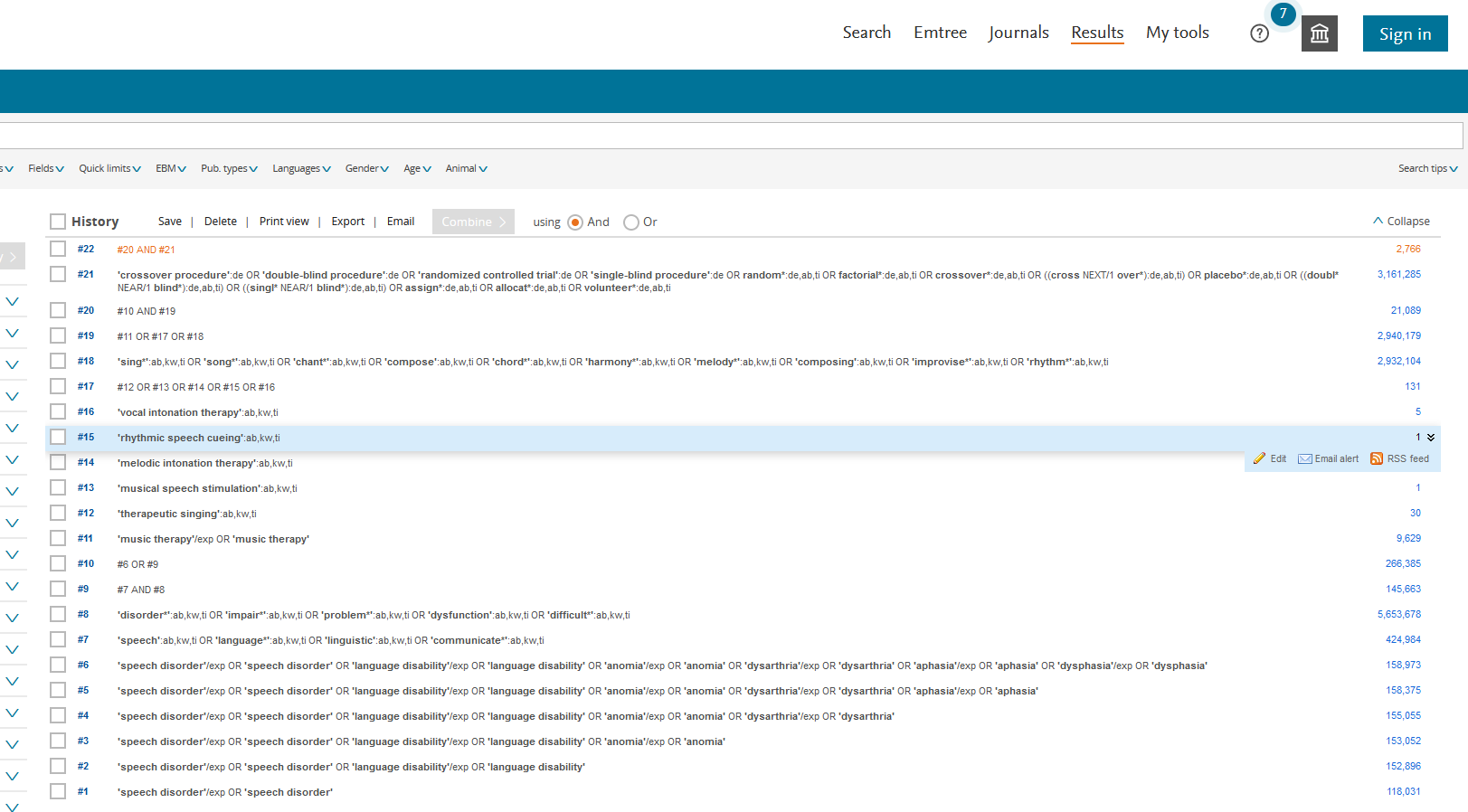


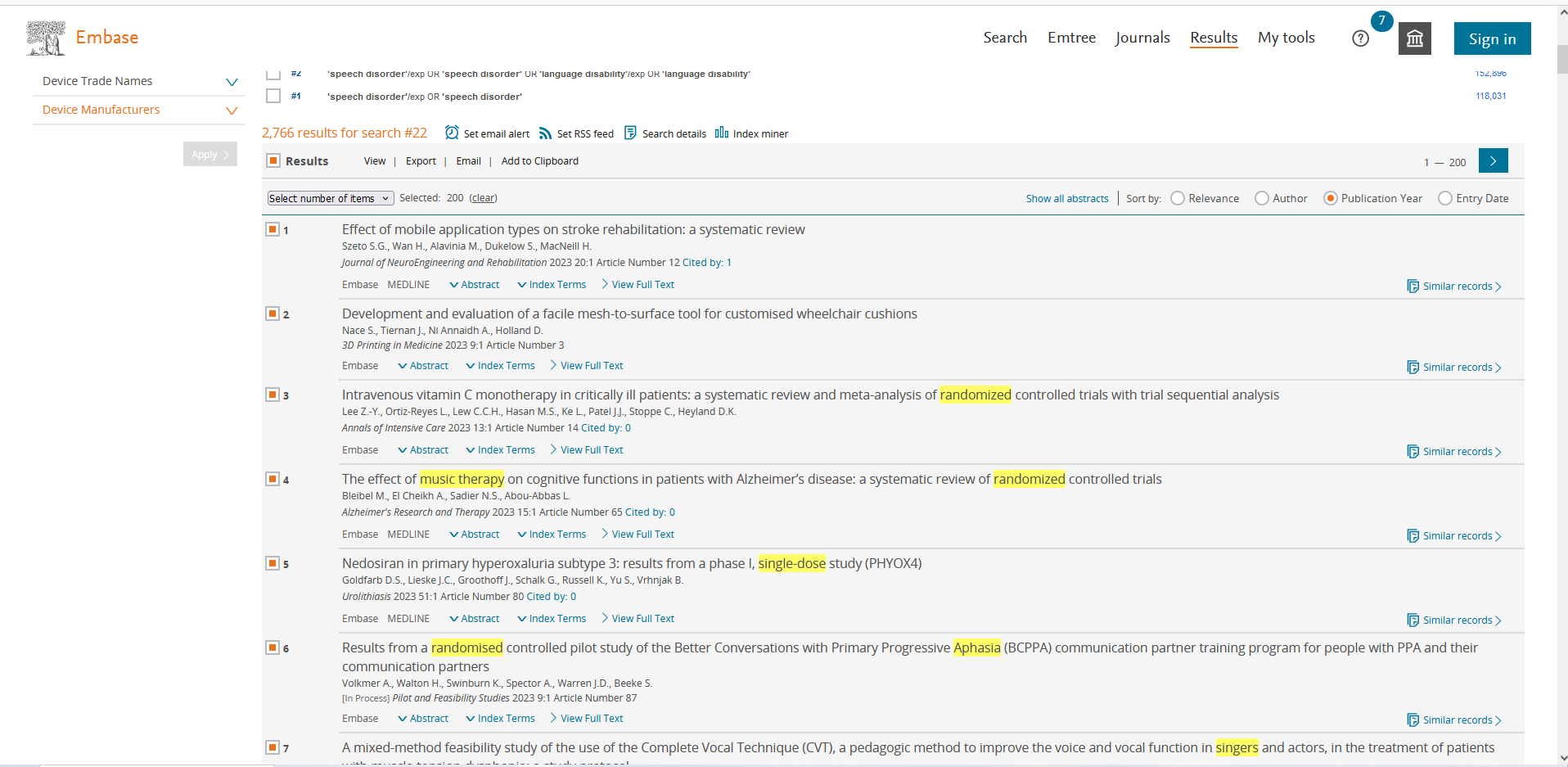

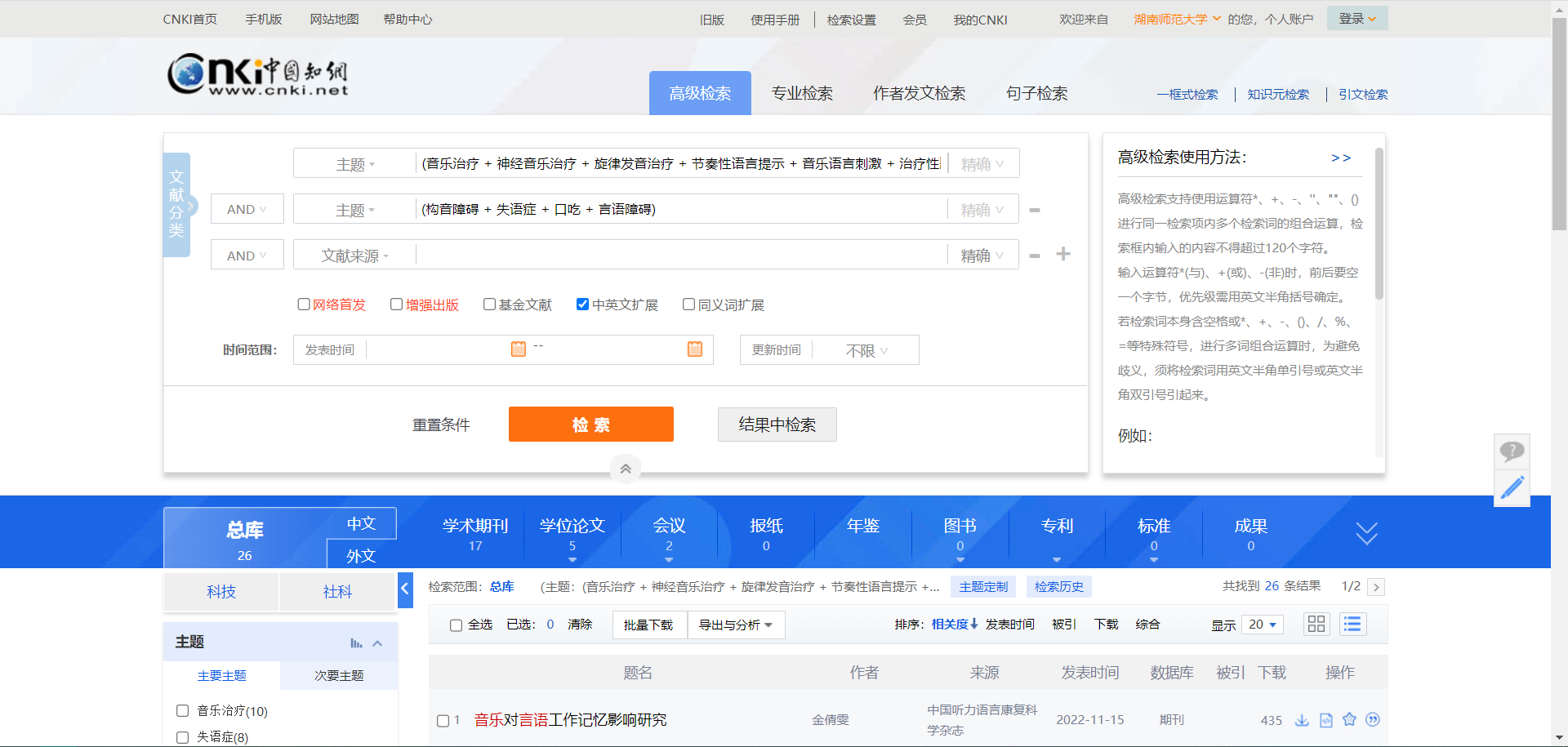

Supplement: Supplementary file 1 [file Table_1.DOC]
