# Supplementary material for: Neurologic music therapy for non-fluent aphasia: a systematic review and meta-analysis of randomized controlled trials
Source: Front Neurol. 2024 May 23;15:1395312. doi: 10.3389/fneur.2024.1395312 (PMC11153767; doi:10.3389/fneur.2024.1395312)
Supplement: Supplementary file 3 [file Table_3.docx]

**Supplementary material 3**

**The list of studies that were excluded after refined screening**

**Unable to get full text (n = 20)**

1 Adaptation of Melodic Intonation Therapy to a Tone Language: A Pilot Study of Tone-Rhythmic Therapy in Mandarin Chinese

Abstract

Introduction: Although melodic intonation therapy (MIT) has proven effective in individuals with non-fluent aphasia in a variety of western languages, its application to Mandarin-speaking aphasic patients has not been thoroughly studied. The adaptation is complicated because Mandarin Chinese is a tone language with specific prosodic elements that differ from Indo-European languages. This study developed a Chinese-specific variant of MIT, i.e., tone-rhythmic therapy (TRT), and tested its efficacy in individuals with non-fluent aphasia. Methods: Six non-fluent aphasic patients were recruited; all of them were admitted to the study over 6 months after stroke and had received a standard program of language therapy. In the current research, tone and rhythmic practice were incorporated into the training procedures, and the adaptation was then examined in patients. The TRT treatment lasted 6 weeks, with five 50-min sessions per week. The Boston Diagnostic Aphasia Examination (BDAE) and the Functional Assessment of Communication Skills for Adults (FACS) tests were used to measure the change in the speech and language skills of patients. Results: The results showed that the patients had increased BDAE and FACS scores after intervention, and the treatment effect lasted for 6 months. Discussion: The modified MIT proved effective for Mandarin-speaking patients with non-fluent aphasia with lasting effects. Further studies evaluating its efficacy are needed for other types of aphasia and other tone languages.

Keywords: Melodic intonation therapy, Tone-rhythmic therapy, Tone language, Aphasic patients

Wenjun Chen, Qian Qian, Jeroen van de Weijer, Shuangshuang Zhu, Manna Wang; Adaptation of Melodic Intonation Therapy to a Tone Language: A Pilot Study of Tone-Rhythmic Therapy in Mandarin Chinese. Folia Phoniatr Logop 3 April 2023; 75 (2): 104–116. [https://doi.org/10.1159/000527225](https://doi.org/10.1159/000527225)

2 {Reference Type}: Journal Article

{Author}: ChiCTR

{Year}: 2023

{Title}: A clinical study on the treatment of chronic post-stroke Broca's aphasia with ''five tone'' therapy based on melody intonation training mode

{URL}: https://www.cochranelibrary.com/central/doi/10.1002/central/CN-02570699/full

{Abstract}: INTERVENTION: Treatment group:''five tones'' therapy based on melodic intonation training model; Conventional speech therapy; Conventional medical treatment; Health education;Control group:Conventional speech therapy; Conventional medical treatment; Health education; CONDITION: Post \stroke aphasia PRIMARY OUTCOME: the Western Aphasia Battery (WAB); SECONDARY OUTCOME: the Communicative Ability Scale (CADL);the percentage of correct information units (CIUs %);the number of correct information units per minute (CIUs / min);electroencephalography (EEG);magnetic resonance imaging (MRI); INCLUSION CRITERIA: 1. Consistent with the diagnosis of stroke in Chinese and Western medicine, and confirmed by craniocerebral CT or MRI examination, the stroke belongs to the left hemisphere of the brain; 2. Broca Aphasia was diagnosed by Western Aphasia Battery (WAB) with BDAE grades 1 \5; 3. Course of disease > 6 months (chronic); 4. Open your right hand; 5. First onset of disease; 6. Aged 45 \75 years; 7. Stable vital signs, clear consciousness, no obvious audio \visual impairment; 8. Passed the ethics review, and the patient voluntarily signed the informed consent, able to understand and cooperate with the rehabilitation evaluation and treatment of this experiment.

3 {Reference Type}: Journal Article

{Author}: NCT

{Year}: 2018

{Title}: Choir Singing in Aphasia Rehabilitation

{Abstract}: BACKGROUND: Singing is a highly stimulating and versatile activity for the brain, combining vocal \motor, auditory, linguistic, cognitive, emotional, and social brain processes, both in the left and right hemisphere. The capacity to sing is often preserved in aphasia after stroke, and singing \based methods, such as Melodic Intonation Therapy (MIT), can be effective in rehabilitating speech production in aphasics. Also emotionally and socially, singing could provide a powerful alternative channel for aphasic patients to express their emotions and interact with others, but the communal or group \level use of singing in aphasia rehabilitation has not been systematically studied. AIMS: The purpose of the study is to determine the clinical and neural efficacy of a novel choir singing intervention in subacute/chronic aphasia. Specifically, the targeted outcomes are (i) verbal and vocal \motor skills, (ii) cognitive skills, (iii) emotional functioning and quality of life, (iv) caregiver psychological well \being, and (v) structural and functional neuroplasticity. In addition, the capacity of singing and music learning in aphasia is explored. METHODS: Subjects are 60 stroke patients with at least minor aphasia (6 months post \stroke) and their family members (FMs, N = 60) from Helsinki area recruited to a cross \over RCT study. Participants are randomized to two groups [N = 60 in both (30 patients, 30 FMs)], which receive a 16 \week choir intervention either during the first (AB group) or second (BA group) half of the follow \up. The intervention is a combination of group training, which utilizes a novel combination of traditional senior choir singing and MIT \like speech training protocols, and home training in which the choir material is trained with a tablet computer. All patients are evaluated at baseline, 5 \month, and 9 \month stages with language, cognitive, and auditory \music tests and questionnaires. Half of the patients (N = 30) also undergo electroencephalography (EEG) and structural and functional magnetic resonance imaging (s/fMRI). FMs are evaluated with questionnaires.

4 {Reference Type}: Journal Article

{Author}: TCTR

{Year}: 2014

{Title}: Effect of Melodic Intonation Therapy on Scoring of Spontaneous Speech, Repetition and Naming in Thai Non-fluent Aphasic Patient

{URL}: https://www.cochranelibrary.com/central/doi/10.1002/central/CN-01879269/full

{Abstract}: INTERVENTION: Thai non \fluent aphasic participant received MIT by trainer for 7 weeks, 5 day/weeks; 1 hour /day, 30 minute in the morning and 30 minute in the evening CONDITION: Stroke, Aphasia, , Melodic Intonation Therapy Thai non \fluent aphasic ; Stroke, Aphasia, , Melodic Intonation Therapy PRIMARY OUTCOME: scoring of spontaneous speech, repetition and naming [Timeframe scoring of spontaneous speech, repetition and naming] SECONDARY OUTCOME: scoring of spontaneous speech, repetition and naming [Timeframe scoring of spontaneous speech, repetition and naming] INCLUSION CRITERIA: non fluent aphasic Age 35 60 years old

5 {Reference Type}: Journal Article

{Author}: NTR

{Year}: 2009

{Title}: Effectiviteit van de Melodic Intonation Therapy (MIT)

{URL}: https://www.cochranelibrary.com/central/doi/10.1002/central/CN-01824325/full

{Abstract}: INTERVENTION: Melodic Intonation Therapy (MIT): Language production therapy in which the melodic aspects of language (rythm, intonation) are used to train the production of sentences. MIT is given for 6 weeks, 5 hour per week. Non \MIT (control condition in post \acute group): Comprehension and production of written sentences. In the control condition, therapy is also given for 6 weeks, 5 hours per week. No treatment (control condition in chronic group): No individual treatment for 6 weeks. Participation in group treatment (1 time per week) is allowed. CONDITION: ; Aphasia, Verbal apraxia ; ; PRIMARY OUTCOME: CIU's/minute on the Sabadel:; ; 1. CIU (correct information units): Words in the patients' production that are adequate, comprehensible, relevant, and informative in relation to the target story;; ; 2. In the Sabadel task, the examiner reads a story to the patient. The story is supported by pictures. The patient's task is to retell the story, while looking at the pictures. SECONDARY OUTCOME: 1. Repetition of trained and untrained items; ; ; 2. AAT (interview, repetition, picture description); ; ; 3. ANELT. INCLUSION CRITERIA: 1. Aphasia after LH stroke; 2. Time post onset: 2 \3 months (post \acute group) or > 1 year (chronic group); 3. Native speaker of Dutch; 4. Candidate for MIT: A. Nonfluent (< 50 words per minute); B. Severe restriction of repetition (AAT subscore repetition <75 and AAT subtest repetition of sentences <11); C. Articulation problems (AAT spontaneous speech score articulation <3); D. Good to moderate auditory comprehension (functional comprehension >5 and AAT subtest auditory comprehension > 32). 5. Age: 18 \80 years; 6. Right \handed.

6 {Reference Type}: Journal Article

{Author}: Jungblut, M.; Aldridge, D.

{Year}: 2004

{Title}: Effects of a specific music therapy approach in the treatment of patients suffering from chronic nonfluent aphasia

{URL}: https://www.cochranelibrary.com/central/doi/10.1002/central/CN-00885476/full

{Volume}: 10

{Issue}: 2

{Pages}: 69 \78

{Notes}: Neurologie und rehabilitation

Journal article

7 {Reference Type}: Journal Article

{Author}: NCT

{Year}: 2020

{Title}: Melodic Intonation Therapy for Tone Language Speakers

{URL}: https://www.cochranelibrary.com/central/doi/10.1002/central/CN-02079172/full

{Tag}: 5

{Star}: 0

{Date Displayed}: 2020-01-01

{Date}: 2020-01-01

{Keywords}: Communication Disorders; Dementia

{Abstract}: One of the traditional therapies for restoring the ability of speech in aphasic patients is Melodic Intonation Therapy (MIT), in which everyday phrases are taught in a singing \like manner. The suggested mechanism for speech recovery is that because of the sharing of brain resources for language and music, the regions normally reserved for singing can be trained to help compensate the speech functions originally subserved by the damaged regions. However, this therapy has primarily been applied to speakers of non \tone languages, in which prosodic features carry a more important role than pitch features in conveying meanings. It remains unknown whether MIT will be equally applicable for speakers of tone languages, in which pitch features likely play a more important role. Another uncertainty concerns whether the efficacy of MIT can be extended to patients with expressive speech impairment due to dementia. This pilot study aims to find out the efficacy of MIT for speech \impaired dementia patients in different verbal tasks. The results of this study will provide preliminary empirical evidence to establish the utility of MIT for Cantonese speakers in Hong Kong.

8 {Reference Type}: Journal Article

{Author}: Tseng, C-E; Lin, C-P; Tsai, P-C; Yip, B-S; Lin, C-M; Yang, F-P

{Year}: 2014

{Title}: Melodic intonation therapy in stroke patients with aphasia: a DTI study

{URL}: https://www.cochranelibrary.com/central/doi/10.1002/central/CN-01067542/full

{Tag}: 5

{Star}: 0

{Volume}: 38

{Issue}: Suppl 1

{Pages}: 40

9 {Reference Type}: Journal Article

{Author}: NCT

{Year}: 2009

{Title}: Melodic-Intonation-Therapy and Speech-Repetition-Therapy for Patients With Non-fluent Aphasia

{URL}: https://www.cochranelibrary.com/central/doi/10.1002/central/CN-01598503/full

{Tag}: 5

{Star}: 0

{Date Displayed}: 2009-01-01

{Date}: 2009-01-01

{Keywords}: Aphasia; Aphasia, Broca; Cerebral Infarction; Infarction; Stroke

{Abstract}: We are doing this clinical trial in order to evaluate two different treatments for non \fluent aphasia: Melodic Intonation Therapy (MIT) and Speech Repetition Therapy (SRT). MIT uses a simple form of singing, while SRT uses intensive repetition of a set of words and phrases. We want to see which intensive form of treatment is more effective in leading to an improvement in speech output compared to a no \therapy control period, and whether either treatment can cause changes in brain activity during speaking and changes in brain structure. We will use a technique known as functional Magnetic Resonance Imaging (fMRI) to measure blood flow changes in the brain and structural MRI that assess brain anatomy and connections between brain regions. We will use fMRI to assess brain activity while a patient speaks, sings, and hums. We will assess changes in brain activity and in brain structure by comparing scans done prior to treatment to scans obtained after treatment and we will also examine changes between treatment groups. We will correlate changes in brain activity and brain structure with changes in language test scores.

10 {Reference Type}: Journal Article

{Author}: Jungblut, M.

{Year}: 2005

{Title}: Music Therapy for People with Chronic Aphasia: a Controlled Study

{URL}: https://www.cochranelibrary.com/central/doi/10.1002/central/CN-00690858/full

11 {Reference Type}: Journal Article

{Author}: ACTRN

{Year}: 2016

{Title}: Parkinsons disease: a voice, singing and music study

{URL}: https://www.cochranelibrary.com/central/doi/10.1002/central/CN-02441723/full

{Tag}: 5

{Star}: 0

{Date Displayed}: 2016-01-01

{Date}: 2016-01-01

{Abstract}: INTERVENTION: Voice and Choral Singing Group Therapy. Group sessions will be led by an experienced clinician Speech Language Therapist and musician. Activities will consist of respiratory, phonatory, oro \motor and relaxation exercise and singing a selection of songs. The group sessions will take place once a week for one and a half hours weekly over 9 continuous weeks. Adherence will be monitored using a register of attendance at each session. CONDITION: Parkinson's disease PRIMARY OUTCOME: Maximum phonation time (secs) ; Voice samples captured for MPT will be edited and analysed using the Multi Dimensional Voice Programme. ; The recordings (44.1 kHz sampling rate, 16 bit) will be captured using an AKG C410 (Harman International, Austria) head mounted condenser microphone connected to a 24 \bit/96kHz audio interface with preamp (M \Audio Mobile Pre USB) and recorded digitally. Acoustic analysis of all the speech samples will be completed using Sona \Speech II 'Trademark' Software (KayPENTAX). ; Voice amplitude ; Voice/speech levels will be measured using a sound level meter (SLM) (CEL \244 Digital Integrating Sound Level Meter Class 2, Casella). ; Three acoustic values: ; 1. Slow, C \weighted sound level (LCS) ; 2. Average, C \weighted sound level (Lceq) ; 3. Maximum sound level (Lmx) ; Voice quality (jitter, Shim) ; Voice samples captured for voice quality will be edited and analysed using the Multi Dimensional Voice Programme. ; Programme (MDVP). Spectral analysis will include: ; 1. Average Fundamental Frequency (Ave F0) ; 2. Relative Average Perturbation (RAP) ; 3. Shimmer % \Shim (Shim) ; 4. Fundamental Frequency Variation (vF0) ; ; As well as these data, further data analysis of prosody will include: ; 1. Mean Fundamental Frequency (MeanF0) in Hertz (Hz) ; 2. Standard Deviation (SD) ; 3. Variance in Fundamental Frequency (vF0) ; 4. Semi \tones (STR) ; 5. Standard Deviation Semitone (SDS) ; ; The recordings and spectral analysis using (44.1 kHz sampling rate, 16 bit) will be captured using an AKG C410 (Harman International, Austria) head mounted condenser microphone connected to a 24 \bit/96kHz audio interface with preamp (M \Audio Mobile Pre USB) and recorded digitally. Acoustic analysis of all the speech samples will be completed using Sona \Speech II 'Trademark' Software (KayPENTAX). ; SECONDARY OUTCOME: ADLs/functional Motor and Non \Motor ; This is a composite outcome measure. Using the MDS \UPDRS Patient self \evaluation questionnaire ; Part I: Non \Motor Aspects of Experiences of Daily Living (nM \EDL) ; Part II: Motor Aspects of Experiences of Daily Living (M \EDL) ; Cognitive Impairment ; Using the Addenbrookes Cognitive Examination ACE \III (NZed) Depression Anxiety ; Using Depression Anxiety Stress Scales DASS \21 Participant questionnaire ; This is a composite outcome measure. To determine participant views on enjoyment, engagement, meaningfulness of participation. Questionnaire adapted from (Fogg & Talmage 2011). Quality of Life ; Using Parkinson s disease quality of life Scale PDQ \8 This is a composite primary outcome. ; Using the Voice Handicap Index ; Respiration and subglottic pressure will also be measured using a Phonatory Aerodynamic System (PAS) which calculates: Average phonatory flow rate, Vital capacity, Glottal resistance and Subglottal pressure. Voice VHI \10 + VHI \10P INCLUSION CRITERIA: All participants will have idiopathic Parkinson s disease diagnosed by a Consultant Neurologist and will not have any other neurological disorder other than PD. Participants will have no singing or VCST in the previous 12 months before the study treatment period.

12 {Reference Type}: Journal Article

{Author}: ChiCTR

{Year}: 2020

{Title}: Research on the clinical and mechanism of melodic intonation therapy to non-fluent aphasia after stroke

{URL}: https://www.cochranelibrary.com/central/doi/10.1002/central/CN-02437147/full

{Abstract}: INTERVENTION: Intervention group:Melodic Intonation Therapy;Control group:Speech Therapy; CONDITION: Cerebrovascular disease PRIMARY OUTCOME: curative effect; INCLUSION CRITERIA: 1. From September 2020 to June 2021, 40 patients with non fluent aphasia after stroke were selected in our hospital. 2. INCLUSION CRITERIA: left cerebral infarction or intracerebral hemorrhage; meeting the diagnostic criteria of non fluent aphasia; less active language and less fluent expression; aged from 18 to 75 years old; fair hearing, with certain understanding and willingness to express; good cooperation, stable mood and good attention; tolerance to sitting training; patients and their families know and agree with this study.

13 {Reference Type}: Journal Article

{Author}: NCT

{Year}: 2020

{Title}: Rhythm-based Intervention in Aphasia

{URL}: https://www.cochranelibrary.com/central/doi/10.1002/central/CN-02181896/full

{Tag}: 5

{Star}: 0

{Date Displayed}: 2020-01-01

{Date}: 2020-01-01

{Keywords}: Aphasia

{Abstract}: Every year, approximately 800,000 people have a stroke in the United States alone. Among these individuals, roughly 100,000 are diagnosed with aphasia \a disorder characterized by profound challenges in daily communication with their families and peers. Notably, many individuals with aphasia can sing despite their speech difficulties, an observation which led to the development of melodic \intonation therapy (MIT) in the 1970s. Although MIT has since been accepted as a viable aphasia therapy by the American Academy of Neurology, the underlying neurological mechanisms that enable speech recovery remain poorly understood. Here, we propose a highly interdisciplinary approach to study the neural mechanisms of language recovery in aphasia through a novel rhythm \based language intervention. Our ultimate goal is to help clinicians and therapists optimize aphasia treatment by elucidating the neural basis underlying music \induced language recovery through multimodal neuroimaging and novel statistical analysis. In particular, we will test a hypothesis that rhythm alone is sufficient to facilitate language recovery, without melody To explore this hypothesis, we recently devised a novel framework for music \based language therapy that solely leverages rhythm to facilitate language production at the phrase or sentence level. Our therapy was used in a case study with a patient with chronic aphasia who had severely impaired speech fluency due to a large unilateral stroke in the left hemisphere. Following eight weeks of rhythm therapy, she exhibited remarkable improvement in speech production (i.e., from 1 \2 spontaneous words to 16 sentences made from 42 words), and increased functional and structural connectivity within key regions of interest associated with the right sensorimotor network. While compelling, these preliminary data warrant further validation using proper active controls and a larger sample size, a goal to be achieved through the proposed research. We have since translated the rhythm therapy into a fun and engaging game termed "TheraBeat" that can be installed on a tablet PC or smartphone. In the proposed research, we will use TheraBeat as a home \based aphasia therapeutic intervention to minimize the burden of patient travel and increase accessibility to therapy. The proposed study will be accomplished by pursuing the following specific aims:. This prediction is based upon the theoretical and neuroscientific framework that demonstrates how language heavily relies on rhythm processes mediated by the bilateral sensorimotor network. Aim 1. Determine the therapeutic role of rhythm in speech recovery for people with aphasia. Based on recent evidence garnered by our group as well as others, we expect that rhythm \based rehabilitation will enhance speech fluency in people with chronic aphasia, i.e., our target patients who are beyond six months after the onset of stroke. This hypothesis will be tested by directly comparing post \therapy outcomes of the treatment group, who will practice speech production daily through rhythmic activity, to an active control group who will receive conventional speech production therapy without the use of rhythmic activities. By using an innovative rhythm \based therapeutic intervention and cutting \edge neuroimaging techniques, we will address hitherto unknown questions regarding how and why music works as a therapeutic regimen for aphasia rehabilitation. With expertise in aphasia rehabilitation, neuroimaging, computer science, and data analytics, our interdisciplinary research team is well poised to undertake this investigation. The proposed research will serve as a critical stepping stone toward understanding the therapeutic role of music in neurological disorders. Our results will lay the foundations for future studies on music \induced language recovery. Aim 2. Characterize the neural plasticity promoted by rhythm \based intervention. We hypothesize that daily participation in our new therapy program will promote neuroplasticity along the sensorimotor network, especially within the intact perilesional left or right fronto \striatal circuits that are known to play a key role in speech production and fluency. To determine structural neuroplasticity following the intervention, we will utilize patented myelin \based MRI in combination with diffusion tensor imaging (DTI). To explore functional neuroplasticity, we will use both resting \state fMRI and functional near \infrared spectroscopy (fNIRS). These structural and functional data will be analyzed via a novel statistical shape analysis that captures morphological changes, which cannot be detected by simple statistical approaches.

14 {Reference Type}: Journal Article

{Author}: Tarrant, M.

{Year}: 2018

{Title}: Singing for People With Aphasia (SPA)

{URL}: https://www.cochranelibrary.com/central/doi/10.1002/central/CN-01659037/full

{Tag}: 5

{Star}: 0

{Date Displayed}: 2018-01-01

{Date}: 2018-01-01

{Notes}: Journal article

15 {Reference Type}: Journal Article

{Author}: UMIN

{Year}: 2014

{Title}: The effect of Japanese version of melodic intonation therapy to aphasia: an fMRI study

{URL}: https://www.cochranelibrary.com/central/doi/10.1002/central/CN-01824502/full

{Tag}: 5

{Star}: 0

{Date Displayed}: 2014-01-01

{Date}: 2014-01-01

{Abstract}: INTERVENTION: Speech training using Japanese version of MIT CONDITION: patients with aphasia PRIMARY OUTCOME: Western Aphasia Battery (WAB) SECONDARY OUTCOME: Images of fMRI INCLUSION CRITERIA: 1.Over 6 months after the onset of aphasia 2.Stable physical and mental condition to do the speech training

{Notes}: https://trialsearch.who.int/Trial2.aspx?TrialID=JPRN-UMIN000013991

Trial registry record

16 {Reference Type}: Journal Article

{Author}: Cohen, N. S.

{Year}: 1992

{Title}: The effect of singing instruction on the speech production of neurologically impaired persons

{URL}: https://www.cochranelibrary.com/central/doi/10.1002/central/CN-00371128/full

{Tag}: 5

{Star}: 0

{Volume}: XXIX

{Issue}: 2

{Pages}: 87 \102

{Date Displayed}: 1992-01-01

{Date}: 1992-01-01

{Notes}: Journal of music therapy

Journal article

17 {Reference Type}: Journal Article

{Author}: DRKS

{Year}: 2021

{Title}: The efficacy of a directed rhythmic-melodic voice training in the treatment of chronic non-fluent aphasia -Behavioral and imaging results-

{URL}: https://www.cochranelibrary.com/central/doi/10.1002/central/CN-02350439/full

{Tag}: 5

{Star}: 0

{Date Displayed}: 2021-01-01

{Date}: 2021-01-01

{Abstract}: INTERVENTION: Intervention 1: The patient group received 32 individual therapies of targeted rhythmic \melodic voice training. Intervention 2: The control group received 32 individual sessions of speech therapy with a focus on improving expressive language performance. CONDITION: I60 \I69 \ Cerebrovascular diseases PRIMARY OUTCOME: Measurement of language performance using the Aachen Aphasia Test, a standardized language test. SECONDARY OUTCOME: Measurement of changes in brain activity using fMRI. INCLUSION CRITERIA: chronic aphasia (time poststroke: at least 1 year), after a first \ever left \hemisphere stroke, non \fluent aphasia (global aphasia or Broca s aphasia) according to the ALLOC classification procedure (Habbema, Hermans & van den Broeck, 1974), premorbidly right \handed as determined by means of the Edinburgh Handedness Inventory (Oldfield, 1971), native language: German, sufficient auditory comprehension to understand the instructions, consent to participating in 32 single therapy sessions over a period of four months after random allocation to either of the two groups, consent to participating in language tests (AAT) before and after therapy, consent to refraining from any other language or cognitive training during participation in the study

{Notes}: https://trialsearch.who.int/Trial2.aspx?TrialID=DRKS00026730

Trial registry record

18 {Reference Type}: Journal Article

{Author}: van der Meulen, A. C.

{Year}: 2009

{Title}: The efficacy of Melodic Intonation Therapy (MIT) in aphasia rehabilitation

{URL}: https://www.cochranelibrary.com/central/doi/10.1002/central/CN-00776592/full

{Tag}: 5

{Star}: 0

{Date Displayed}: 2009-01-01

{Date}: 2009-01-01

{Notes}: Nederlands trial register (http://www.trialregister.nl)

Journal article

19 : G. Schlaug, Department of Neurology, Music, Neuroimaging, Beth Israel Deaconess Medical Center, Harvard Medical School, 330 Brookline Avenue, Boston, MA 02215, United States

Journal : Future NeurologyFuture Neurol.

IF : 1.3

Year : 20102023

Tag : 5

Star : 5

Page : 657-665

Place: other

Author : other

Time : 2023/8/18 10:29:00

Type : 9270

Type : JOUR

ISBN/ISSN : 1479-6708

Doi : 10.2217/fnl.10.442010/1/1

20 Type: Journal

Title : Singing to speaking: observations in healthy singers and patients with Broca's aphasia

Author : Schlaug, G.

Year : 20122023

Date : 2023/8/18 10:15:00

Type: 9354

Type: JOUR

other : 2012/1/1

**Not a randomized controlled trial (n =10)**

1 Aleksi J. Sihvonen, 2020 Vocal music enhances memory and language recovery afterstroke: pooled results from two RCTs

2 Tamplin, J.,2019 ParkinSong a Controlled Trial of Singing-Based Therapy for Parkinson's Disease

3 Han, Eun Young，2018 Individual Therapeutic Singing Program for Vocal Quality and Depression in Parkinson's Disease

4 Tamplin, J., 2014 The effect of singing training on voice quality for people with quadriplegia

5 Ashley N. Higgins,2018 The Effects of a Choral Singing Intervention on Speech Characteristics in Individuals With Parkinson’s Disease: An Exploratory Study

6 Borna Bonakdarpour, 2003 Melodic intonation therapy in Persian aphasic patients

7 Bettina Brendel,2013 Effectiveness of metrical pacing in the treatment of apraxia of speech

8 Sarah Curtis,2019 Tap your hand if you feel the beat: differential effects of tapping in melodic intonation therapy

9 Aleksi J. Sihvonen,2021 Vocal Music Listening Enhances Poststroke Language Network Reorganization

10 Fay Fransella, 1965 An experimental analysis OE the Effect of Rhythm on the Speech of Stutterers

**Outcomes do not meet inclusion criteria(n=5)**

1 Zhang, XY., 2022 The Effect of Vocal Intonation Therapy on Vocal Dysfunction in Patients With Cervical Spinal Cord Injury a Randomized Control Trial

2 Mohseni, Z.,2023 Voice improvement following conventional speech therapy combined with singing intervention in people with Parkinson's disease a three-arm randomised controlled trial

3 Icht, M.,2021 Improving speech characteristics of young adults with congenital

4 Katerina Aravantinou-Fatorou,2020 Efficacy of exercise rehabilitation program accompanied by experiential music for recovery of aphasia in single cerebrovascular accidents: a randomized controlled trial

5 Brotons, M., 2000 The impact of music therapy on language functioning in dementia
