# Supplementary material for: Neurologic music therapy for non-fluent aphasia: a systematic review and meta-analysis of randomized controlled trials
Source: Front Neurol. 2024 May 23;15:1395312. doi: 10.3389/fneur.2024.1395312 (PMC11153767; doi:10.3389/fneur.2024.1395312)
Supplement: Supplementary file 4 [file Table_4.docx]

**Supplementary material 4 Intervention Dose Exploration**


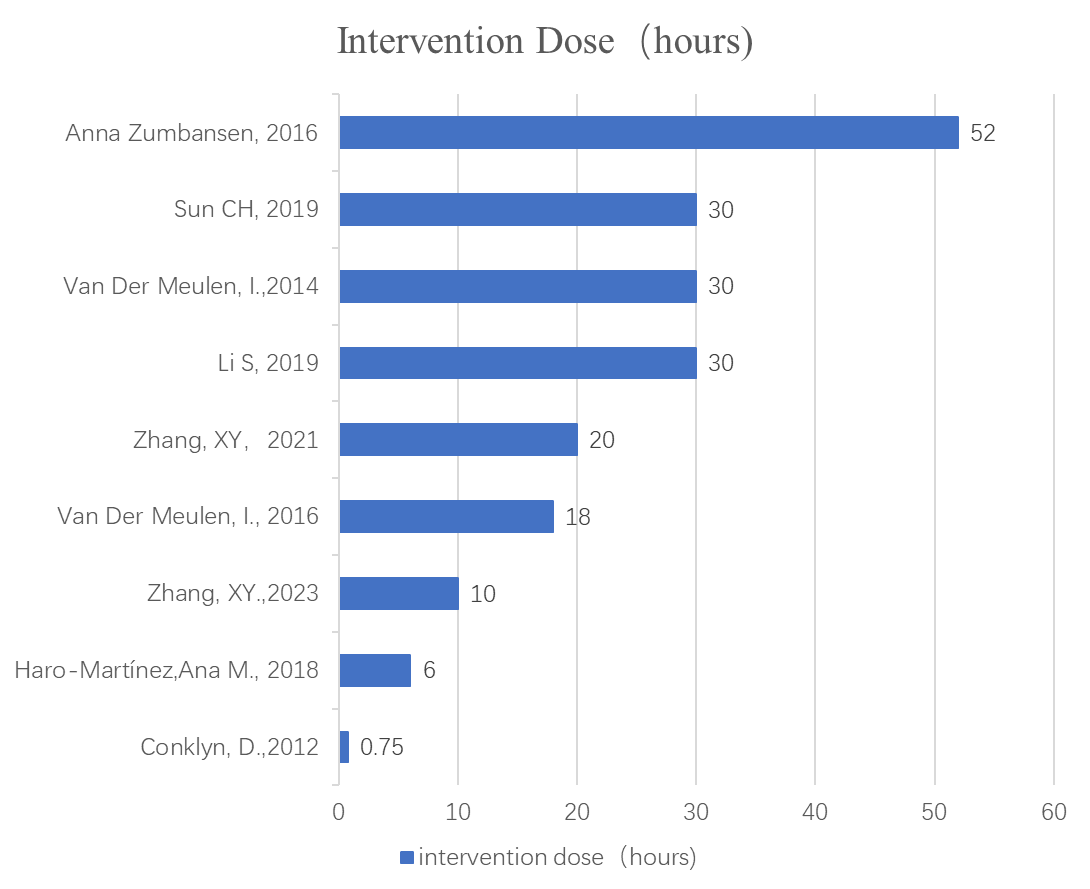


Fig 1 Bar chart of intervention doses of included studies


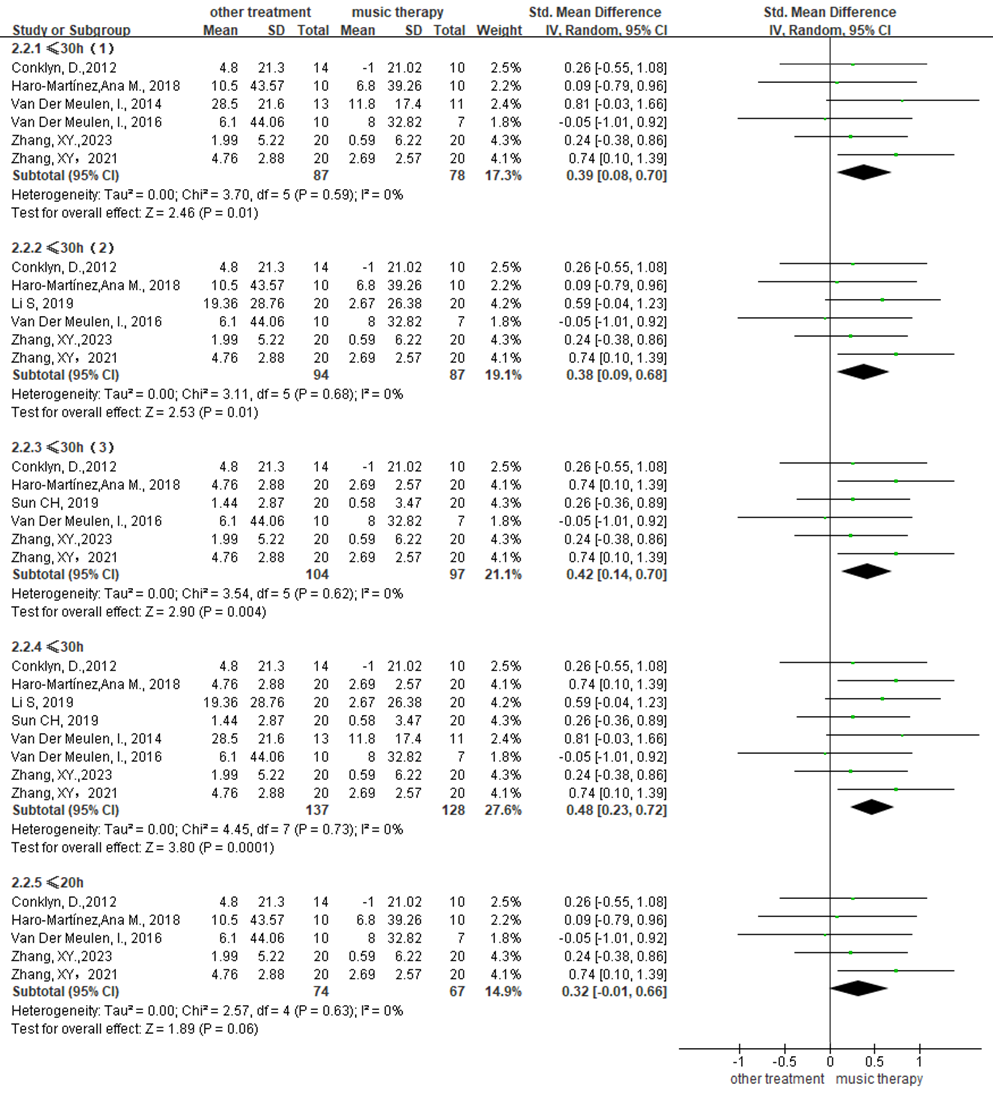
Fig 2 Forest plot of the effect of NMT on Repetition for Exploration of Intervention Dose
