# Supplementary material for: Neurologic music therapy for non-fluent aphasia: a systematic review and meta-analysis of randomized controlled trials
Source: Front Neurol. 2024 May 23;15:1395312. doi: 10.3389/fneur.2024.1395312 (PMC11153767; doi:10.3389/fneur.2024.1395312)
Supplement: Supplementary file 5 [file Table_5.docx]

**Supplementary material 5 Sensitivity Analysis Chart
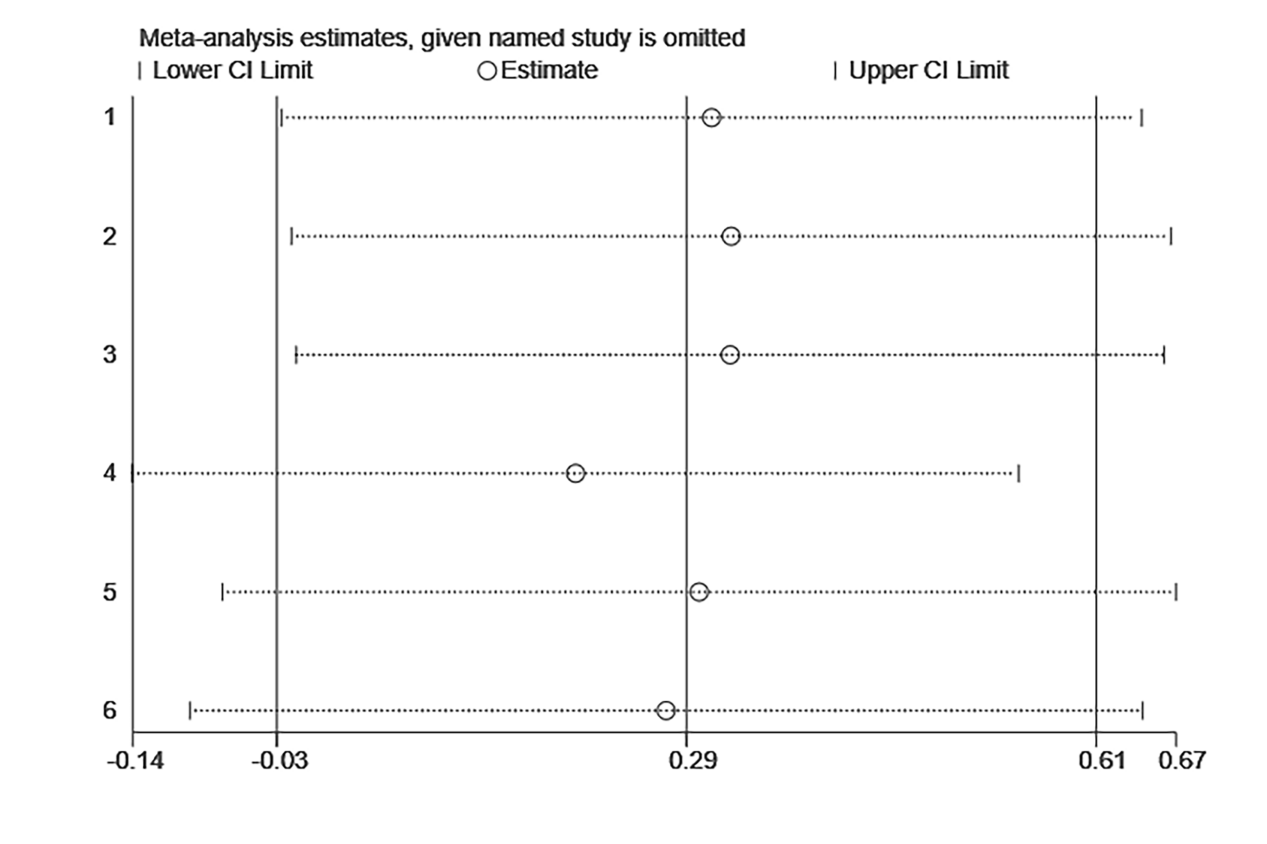
**

Fig 1 Sensitivity analysis chart based on naming

**
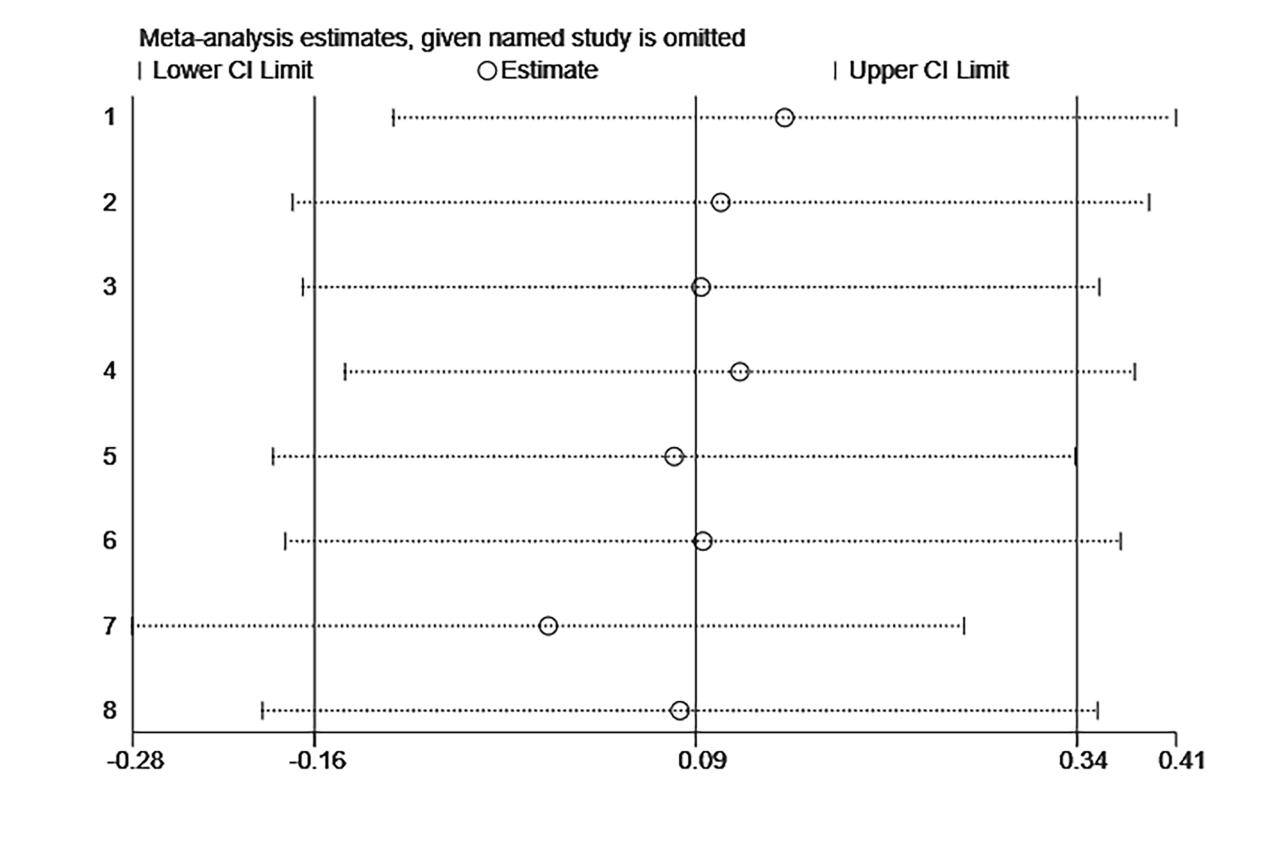
**

Fig 2 Sensitivity analysis chart based on comprehension

**
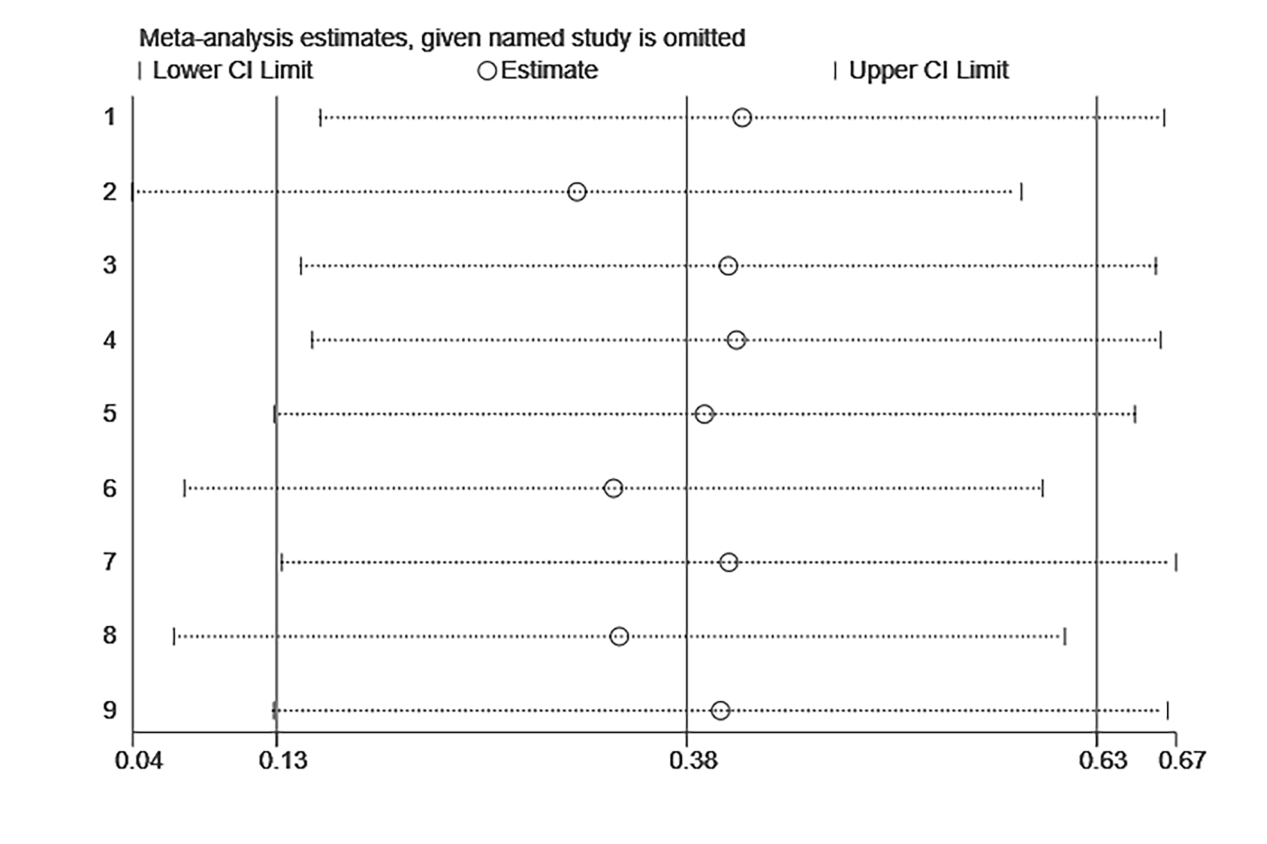
**

Fig 3 Sensitivity analysis chart based on repetition

**
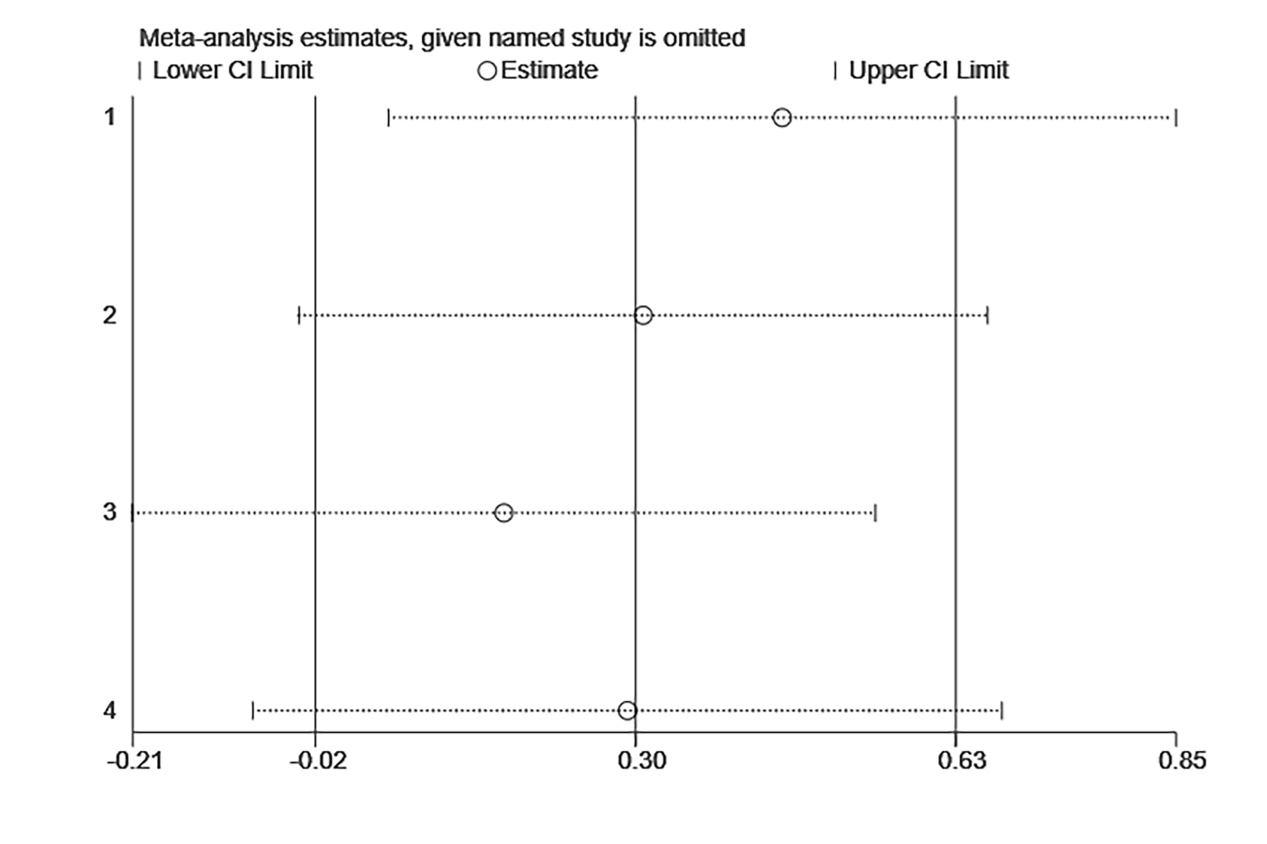
**

Fig 4 Sensitivity analysis chart based on spontanous speech


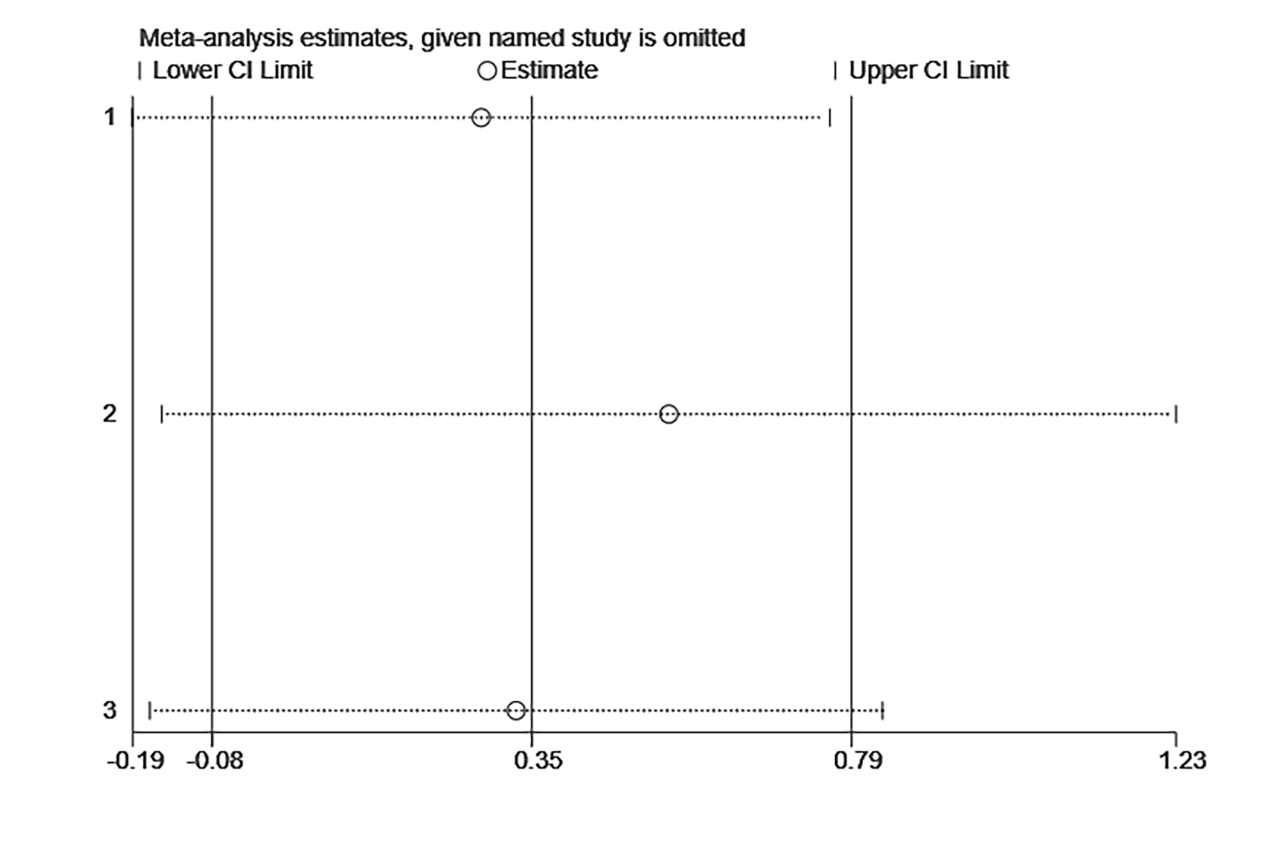


Fig 5 Sensitivity analysis chart based on Communication
